# Enhanced small green fluorescent proteins as a multisensing platform for biosensor development

**DOI:** 10.3389/fbioe.2022.1039317

**Published:** 2022-10-17

**Authors:** Guo-Teng Liang, Cuixin Lai, Zejun Yue, Hanbin Zhang, Danyang Li, Zhong Chen, Xingyu Lu, Liang Tao, Fedor V. Subach, Kiryl D. Piatkevich

**Affiliations:** ^1^ School of Life Sciences, Westlake University, Hangzhou, Zhejiang, China; ^2^ Westlake Laboratory of Life Sciences and Biomedicine, Hangzhou, Zhejiang, China; ^3^ Institute of Basic Medical Sciences, Westlake Institute for Advanced Study, Hangzhou, Zhejiang, China; ^4^ Department of Applied Biology and Chemical Technology, The Hong Kong Polytechnic University, Kowloon, Hong Kong SAR, China; ^5^ School of Basic Medical Sciences, Xi’an Jiao Tong University, Xi’an, Shaanxi, China; ^6^ Key Laboratory of Precise Synthesis of Functional Molecules of Zhejiang Province, School of Science, Instrumentation and Service Center for Molecular Sciences, Westlake University, Hangzhou, Zhejiang, China; ^7^ Complex of NBICS Technologies, National Research Center “Kurchatov Institute”, Moscow, Russia

**Keywords:** fluorescent protein biosensor, hypoxia, copper, live cell imaging microscopy, flavin

## Abstract

Engineered light, oxygen, and voltage (LOV)-based proteins are able to fluoresce without oxygen requirement due to the autocatalytic incorporation of exogenous flavin as a chromophore thus allowing for live cell imaging under hypoxic and anaerobic conditions. They were also discovered to have high sensitivity to transition metal ions and physiological flavin derivatives. These properties make flavin-binding fluorescent proteins (FPs) a perspective platform for biosensor development. However, brightness of currently available flavin-binding FPs is limited compared to GFP-like FPs creating a need for their further enhancement and optimization. In this study, we applied a directed molecular evolution approach to develop a pair of flavin-binding FPs, named miniGFP1 and miniGFP2. The miniGFP proteins are characterized by cyan-green fluorescence with excitation/emission maxima at 450/499 nm and a molecular size of ∼13 kDa. We carried out systematic benchmarking of miniGFPs in *Escherichia coli* and cultured mammalian cells against spectrally similar FPs including GFP-like FP, bilirubin-binding FP, and bright flavin-binding FPs. The miniGFPs proteins exhibited improved photochemical properties compared to other flavin-binding FPs enabling long-term live cell imaging. We demonstrated the utility of miniGFPs for live cell imaging in bacterial culture under anaerobic conditions and in CHO cells under hypoxia. The miniGFPs’ fluorescence was highly sensitive to Cu(II) ions in solution with K_d_ values of 67 and 68 nM for miniGFP1 and miniGFP2, respectively. We also observed fluorescence quenching of miniGFPs by the reduced form of Cu(I) suggesting its potential application as an optical indicator for Cu(I) and Cu(II). In addition, miniGFPs showed the ability to selectively bind exogenous flavin mononucleotide demonstrating a potential for utilization as a selective fluorescent flavin indicator. Altogether, miniGFPs can serve as a multisensing platform for fluorescence biosensor development for *in vitro* and in-cell applications.

## Introduction

Since the development of the first genetically encoded calcium biosensor in 1997 (Miyawaki et al., 1997), fluorescent biosensors have become indispensable molecular tools for real-time bioimaging at the cellular and subcellular levels. Fluorescent biosensors have found wide applications in life science research for cell culture and *in vivo* imaging to detect various physiological ions and analytes including potassium, chloride, protons, magnesium, zinc, ATP, cAMP, etc., as well as physiological processes such as neurotransmission, membrane potential, enzymatic activity, redox potential, etc. (Lin and Schnitzer, 2016; Greenwald et al., 2018; Piatkevich et al., 2019) Most of the currently available biosensors are chimeras of fluorescent proteins (FPs) and sensing protein moiety represented by an analyte binding domain (Palmer et al., 2011; Germond et al., 2016; Terai et al., 2019). Engineering chimeric biosensors requires a creative rational design approach complemented by multistep optimization of amino acid linkers between the domains, binding pocket interactions, and FP fluorescence (Sun et al., 2018; Nasu et al., 2021). From another hand, it was discovered that some FPs and chromoproteins possess high environmental sensitivity under physiological conditions and therefore they can be easily converted into single FP-based biosensors for chloride ions (Galietta et al., 2001; Tutol et al., 2021), pH (Miesenböck et al., 1998; Rajendran et al., 2018), temperature (Deepankumar et al., 2015), membrane potential (Kralj et al., 2012), redox potentioan (Hanson et al., 2004), heavy metal ions (Chapleau et al., 2008; Ravikumar et al., 2016) by introducing only a few mutations. Among environmentally sensitive FPs and chromoproteins, FPs derived from LOV-based flavoproteins found in plants and bacteria are characterized by the smallest molecular size and ability to fluoresce when expressed in bacteria and mammalian cells under low oxygen conditions (Walter et al., 2012; Wang et al., 2017). These FPs autocatalytically incorporate flavins as a chromophore, which are essential metabolites found in abundance in the cytoplasm of bacteria, plants, and mammalian cells (Chapman et al., 2008; Abbas and Sibirny, 2011; Hühner et al., 2015). Oxygen-independent fluorescence enabled imaging of flavin-binding FPs under anaerobic and hypoxic conditions as they do not require molecular oxygen for chromophore formation or incorporation into apoprotein (Drepper et al., 2007; Walter et al., 2012; Mukherjee et al., 2013). This property enabled the applications of flavin-binding FPs as fluorescent tags and biosensors for cellular imaging of hypoxically cultured mammalian cells (Walter et al., 2012), obligate anaerobes (Buckley et al., 2016; Seo et al., 2018), and anaerobic pathogens during host cell infections under physiologically relevant conditions (Choi et al., 2011; Lobo et al., 2011). In addition, their smaller size relative to GFP-like FPs (∼12–16 kDa vs. 25–27 kDa for GFP-like FPs) is beneficial for virus-based gene delivery methods (Konermann et al., 2013; Van Den Wollenberg et al., 2015) and tagging proteins of interest (Chapman et al., 2008; Gawthorne et al., 2012; Emmi Scholz et al., 2013; Gawthorne et al., 2016). Several optimized flavin-binding FPs were shown to have high pH and temperature stability thus enabling their application in a wide range of conditions both in live cells and *in vitro* (Mukherjee et al., 2015; Wingen et al., 2017). More recently, the utility of LOV-based FPs was extended to the detection of heavy metal ions including copper (Zou et al., 2020) and mercury (Ravikumar et al., 2015a) as well as flavin derivatives (Anderson et al., 2020). However, compared to GFP-like FPs, the flavin-binding FPs still have room for development regarding their biophysical and biochemical characteristics, especially intracellular fluorescent brightness and photostability, which would promote their wider adaptation as biosensors (Ozbakir et al., 2019).

In this study, we report the development and characterization of a pair of enhanced 13 kDa (111 aa) green FPs, named, miniGFP1 and miniGFP2, which could serve as perspective templates for biosensor development. Systematic characterization of miniGFPs in bacterial and cultured mammalian cells revealed their beneficial photophysical properties compared to other bright flavin-binding FPs. We demonstrated their utility for fluorescence imaging under hypoxic and anaerobic conditions in cultured mammalian cell lines and *Escherichia coli* bacteria, respectively. We also characterized the fluorescence sensitivity and selectivity of miniGFPs to copper ions and flavin derivatives in solution. We discovered that miniGFPs possessed a micromolar affinity to the reduced form of Cu(I), which makes miniGFPs the first flavin-binding FPs, to our knowledge, reported to have sensitivity to Cu(I). Altogether, these results suggested the potential of miniGFP1/2 as a multi-sensing toolbox for biosensing applications.

## Results

### Development of miniGFPs

**FIGURE 1:**
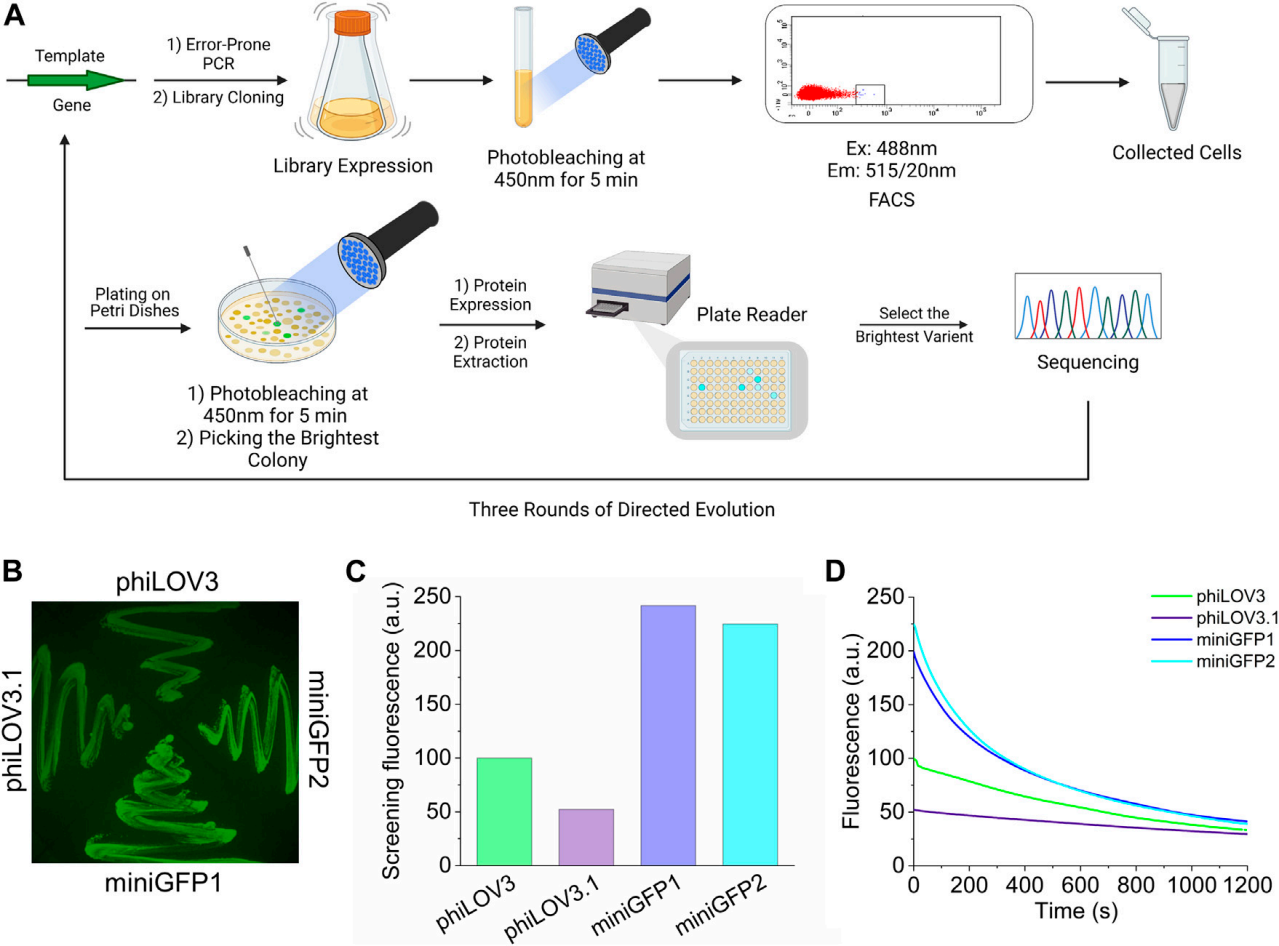
Development of small GFPs. **(A)** Workflow of directed molecular evolution of the flavin-binding miniGFPs in *E.coli*. FACS, fluorescent-activated cell sorting. **(B)** Fluorescence image in the green channel of *E. coli* streaks on LB/agar plate expressing phiLOV3, phiLOV3.1 (an intermediate mutant from the second round of evolution), miniGFP1, and miniGFP2. **(C)** Screening green fluorescence intensity of bacterial streaks shown in **(B)**. **(D)** Screening photobleaching curves for phiLOV3, phiLOV3.1, miniGFP1, and miniGFP2 in *E. coli* under continuous wide-field illumination.

As a starting template, we selected a novel LOV-based flavin-binding FP, called phiLOV3, which was optimized in mammalian cells using a rapid directed molecular evolution approach (Babakhanova et al., 2022). We subjected phiLOV3 to three rounds of directed molecular evolution using *E. coli* as an expression host system (Figure 1A). Bacterial libraries, consisting of 1–10 × 10^6^ independent clones generated by error-prone polymerase chain reaction, were first subjected to 450 nm illumination from an LED to select for photostability followed by screening for the brightest cells in the green channel with a fluorescence-activated cell sorter (FACS; Supplementary Figure S1). The sorted clones were further analyzed using a two-step hierarchical screening strategy, including screening the bacterial colonies under a fluorescent stereomicroscope before and after the 450 nm illumination followed by spectroscopic measurements in solution in a 96-well plate format using a fluorescence microplate reader (Figure 1A, see Supplementary Figure S1 for representative images of bacterial colonies before and after illumination). After each round, the top 5–10 clones were analyzed by sequencing and subjected to the next round of directed molecular evolution. During the selection process, we observed a trade-off between brightness and photostability, for example, intermediate mutant phiLOV3.1 had an improved photobleaching rate but reduced intracellular brightness (Figures 1B–D). After three rounds of directed molecular evolution, we selected two variants, named miniGFP1 and miniGFP2, which exhibited the highest performance under screening conditions quantified as a product of screening brightness measured with a plate reader and photostability on the colonies (each normalized to its maximum value in the group to ensure the roughly equal contribution of each parameter to the product). The miniGFP1 and miniGFP2 proteins were more than 2-fold brighter than their parental protein phiLOV3 when expressed in *E.coli* and retained higher relative brightness after 5 min of the continuous blue light illumination (Figures 1B–D). The miniGFP1 and miniGFP2 proteins had four and five amino acid mutations, respectively, compared to the parental protein phiLOV3 (Supplementary Figure S2). Among introduced mutations, only two common mutations F19S and R90F were located in the β-sheets while other mutations were introduced into the loops between secondary motifs of the proteins (Supplementary Figure S2).

### Spectroscopic and biochemical characterization of miniGFPs

**FIGURE 2:**
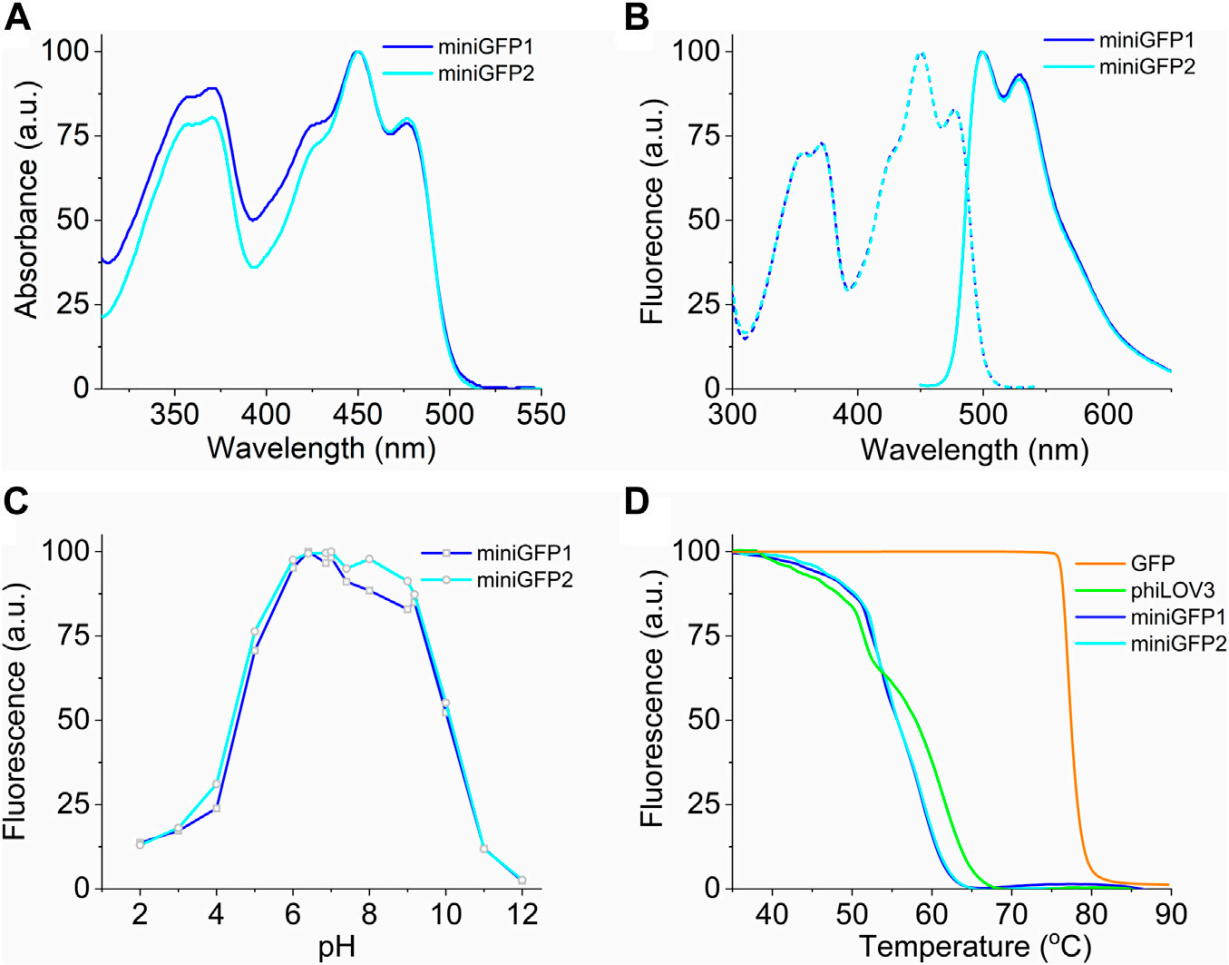
Spectral and biochemical characterization of miniGFP1 and miniGFP2 in solution. **(A)** Absorption spectra of miniGFP1 and miniGFP2. **(B)** Excitation (dashed) and emission (solid) fluorescence spectra of miniGFP1 and miniGFP2. **(C)** Normalized fluorescence of miniGFP1 and miniGFP2 under different pH values. **(D)** Fluorescence thermostability curves for GFP, phiLOV3, miniGFP1, and miniGFP2.

To characterize the spectral and biochemical properties in solution, miniGFPs were expressed in *E.coli* and purified using the standard metal affinity chromatography method. The miniGFP proteins had almost identical absorbance and fluorescence spectra resembling the characteristic spectral profile of flavin-binding FPs (Mukherjee et al., 2013). Absorbance and fluorescence excitation spectra exhibited peaks in the UV-A (350−370 nm) and blue (450 nm) regions of the spectrum, and emission spectra had a wide band with a peak at 499 nm and a prominent shoulder at 525 nm (Figures 2A,B). The molecular brightness of miniGFP1 and miniGFP2 was 1.4-fold higher than that of phiLOV3 mainly due to increased quantum yields (Table 1). The fluorescence lifetime for both miniGFPs was around 4.5 ns (Table 1). The fluorescence of miniGFP1 and miniGFP2 had a bell-shaped pH dependence with the pK_a1_ values of 4.5 and 4.4 and pK_a2_ values of 10.1 and 10.1, respectively (Figure 2C). The thermostability test revealed that miniGFPs had a similar thermostability characterized by T_m_ value of 55.6°C, while their precursor phiLOV3 was slightly more thermostable with T_m_ of 58°C (Figure 2D). For comparison, the T_m_ value for EGFP was about 80°C under identical conditions (Figure 2D).

**TABLE 1:** Properties of the FMN-binding fluorescent proteins, miniGFP1 and miniGFP2.

Protein	Abs (nm)	Ex (nm)	Em (nm)	EC (M^−1^ cm^−1^)	QY (%)	Molecular brightness^a^	Life-time (ns)	pK_a_	Thermo-stability (°C)^b^	Intracellular photostability (s)^c^	References
phiLOV2.1	451/476	451	501	13,500	20	2,700	N.D.	3.0	N.D.	N.D.	Christie et al. (2012), Babakhanova et al. (2022)
phiLOV3	452/477	452	502	15,800	22	3,480	N.D.	3.3	57.8	300	Babakhanova et al. (2022)
CreiLOV	N.D.	448	498/527	N.D.	51	6,375	N.D.	<3.0	N.D.	4	Mukherjee et al. (2015)
BR1	N.D.	449	496	N.D.	45	5,625	N.D.		N.D.	1	Ko et al. (2019)
miniGFP1	450/477	450	499	16,800	31	5,130	4.46	4.5	55.6	197	This study
miniGFP2	450/477	450	499	16,200	32	5,132	4.54	4.4	55.6	207	This study

Abs, Absorbance peaks; Ex, fluorescence excitation peak; Em, fluorescence emission peak; EC, extinction coefficient; QY, quantum yield.

^a^ Molecular brightness is calculated by the extinction coefficient and quantum yield.

^b^ Thermostability is evaluated by the temperature that the fluorescence of FMN-binding proteins receded to its half by heat comparing to its fluorescence at RT.

^c^ Measured in live MEF cells under continuous wide-field excitation (20% of 470 nm SpectraIII Light engine with ×20 NA0.75 objective lens).

**FIGURE 3:**
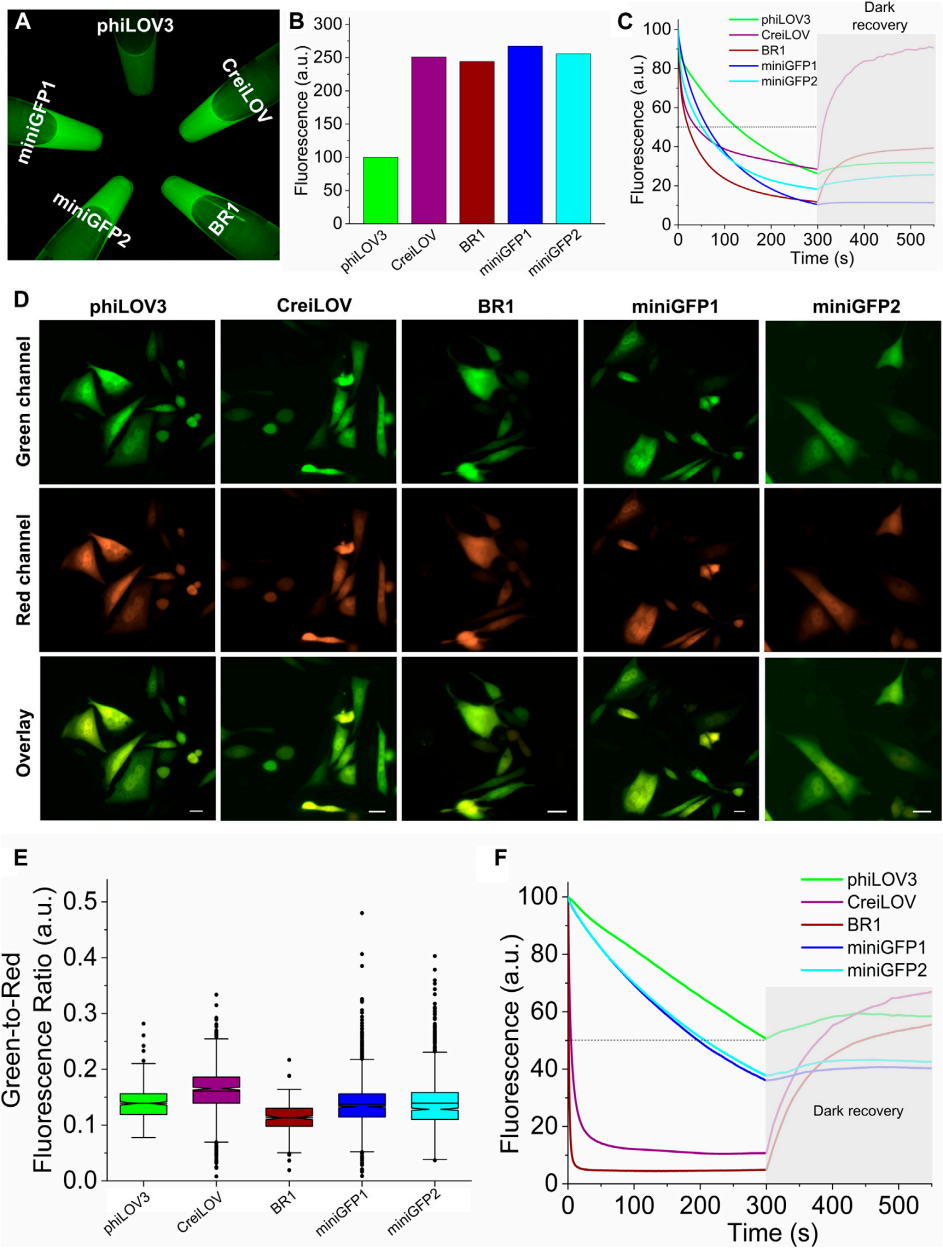
Biochemical characterization of miniGFPs in comparison to phiLOV3, CreiLOV, and BR1. **(A)** Green fluorescence image of crude extract from *E.coli* cells expressing selected proteins. **(B)** Quantification of fluorescence intensity of the samples shown in **(A)**. **(C)** Photobleaching and dark recovery curves measured on bacterial streaks expressing the selected proteins (*n* = 1 streak for each protein; shaded area correspond to dark recovery phase). **(D)** Representative images of CHO cells expressing the selected proteins in green and red channels. Scale bar, 20 µm. **(E)** Green-to-red fluorescence ratios for phiLOV3, CreiLOV, BR1, miniGFP1, and miniGFP2 expressed in CHO cells (*n* = 370, 905, 211, 2,220, 1,501 cells from two independent transfections each, respectively). Box plots with notches are used for data visualization (narrow part of notch, median; top and bottom of the notch, 95% confidence interval for the median; top and bottom horizontal lines, 25% and 75% percentiles for the data; whiskers extend 1.5 × the interquartile range from the 25th and 75th percentiles; horizontal line, mean; dots, outliers). **(F)** Photobleaching and dark recovery curves measured on live CHO cells expressing phiLOV3, CreiLOV, BR1, miniGFP1, and miniGFP2 proteins (*n* = 114, 52, 74, 98, and 62 cells from two independent transfection each, respectively).

Next, we sought to compare intracellular brightness and photostability of miniGFPs to spectrally similar flavin-binding FPs, which are characterized by the highest molecular brightness in the class and comparable molecular size (i.e., <13 kDa). Since our goal was to develop FPs for live cell imaging, we excluded bright flavin-binding proteins that are known to generate singlet oxygen upon illumination, such as miniSOG (Shu et al., 2011). By searching FPbase (https://www.fpbase.org/) (Lambert, 2019), we selected a pair of FPs BR1 (Ko et al., 2019) and CreiLOV (Mukherjee et al., 2015), which reported molecular brightness was higher than that of miniGFPs (Table 1). First, we expressed the proteins in *E. coli* using pBAD vector and measured fluorescence brightness of the crude protein extracts in standard green channel. All proteins exhibited comparable brightness except for phiLOV3, which was about 2.5-fold dimmer (Figures 3A,B). When measuring photostability in *E.coli* cells under continuous wide-field illumination, we also assessed dark recovery as many flavin binding FPs exhibit reversible photoswitching (Christie et al., 2012; Gregor et al., 2018), which is an undesired property for quantitative imaging. The miniGFPs proteins demonstrated superior photostability compared to BR1 and CreiLOV although they were about twice less photostable as phiLOV3 (Figure 3C). However, miniGFP1 showed almost no dark recovery (less than 8% relative to initial brightness), in turn, dark recovery for phiLOV3 and miniGFP2 was slightly higher reaching 21% and 45%, respectively. Recovery of fluorescence post-photobleaching for BR1 and CreiLOV was more than 3-fold relative to the photobleached state. Based on the photophysical properties miniGFP1 was the most appropriate protein for quantitative imaging.

To access the intracellular brightness and photostability in mammalian cells, we co-expressed miniGFPs with bright RFP FusionRed (Shemiakina et al., 2012) *via* the self-cleavable P2A peptide to account for the expression level variations during transient transfection. For comparison, we also expressed phiLOV3, CreiLOV, and BR1 proteins. After transient transfection, live Chinese hamster ovary (CHO) cells were imaged in the standard green and red channels under a wide-field microscope, and green-to-red fluorescence ratios were calculated for quantitative comparison of intracellular brightness. The miniGFPs fluorescence was evenly distributed throughout the cytoplasm of CHO cells without any visible aggregation or organelle accumulation similarly to other FPs used for reference (Figure 3D). Expression of miniGFP1 in cultured mouse neurons also produced easily detectable green fluorescence without signs of aggregation similar to phiLOV3 (Supplementary Figure S3). Quantification of the green-to-red ratios revealed that intracellular brightness of both miniGFPs was comparable to that of phiLOV3 (Figure 3E). In turn, the intracellular brightness of CreiLOV was 15% higher than that of phiLOV3 and the intracellular brightness of BR1 was 19% lower than that of phiLOV3 (Figure 3E). The photobleaching half-times for miniGFP1 and miniGFP2 were 197 s and 207 s vs. ∼300 s for phiLOV3 (Figure 3F). After photobleaching miniGFPs exhibited insignificant dark recovery of less than 6% from the initial level. Dark recovery for phiLOV3 was about 8%. Under the same condition, photobleaching half-times of CreiLOV and BR1 were 4.5 and 1 s reaching minimal fluorescence in less than 100 s of continuous illumination, however, similarly to *E.coli* experiments fluorescence of CreiLOV and BR1 recovered more than 50% from initial level (Figure 3F). These results indicated that miniGFPs were more suitable for quantitative live cell imaging than CreiLOV and BR1, which demonstrated photoswitching behavior.

To further benchmark the performance of miniGFPs, we measured intracellular brightness and photostability in MEF, HEK, CHO, and HeLa cells in comparison with phiLOV3, EGFP, and UnaG (Supplementary Figure S4). Overall, miniGFPs appeared to have about 4- to 8-fold lower brightness than EGFP and UnaG. However, miniGFPs outperformed UnaG in terms of photostability in all tested cell lines, while EGFP remained the most photostable FP except for in HEK cells where phiLOV3 was the most photostable (Supplementary Figure S4). Altogether, miniGFPs were suitable for live cell imaging in different cell types and demonstrated improved photochemical properties compared to other bright flavin-binding FPs.

### Characterization under anaerobic and hypoxic conditions

**FIGURE 4:**
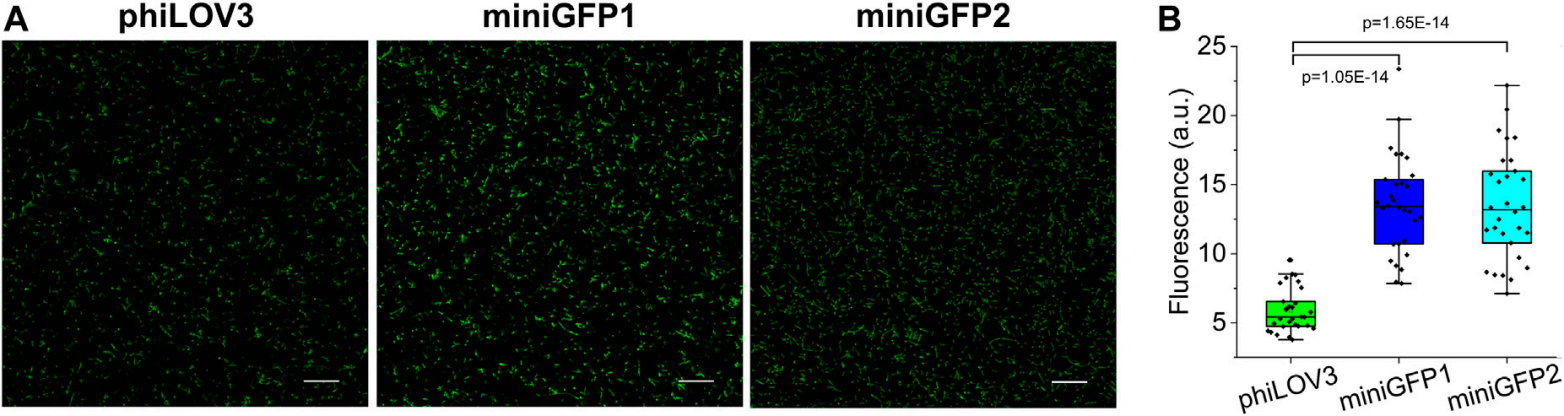
Characterization of miniGFPs under anaerobic conditions. **(A)** Fluorescence confocal images of anaerobically cultured *E. coli* cells expressing phiLOV3, miniGFP1, and miniGFP2. Scale bar, 20 µm. **(B)** Quantified fluorescence of *E. coli* expressing proteins shown in **(A)** (P_miniGFP1-phiLOV3_ = 1.05E-14, P_miniGFP2-phiLOV3_ = 1.65E-14, ANOVA—Fisher Test).

One of the most distinctive features of flavin-binding FPs is their ability to fluorescence in oxygen-free conditions. To this end, we tested the utility of miniGFPs for live cell imaging under anaerobic and hypoxic conditions. First, we transformed the miniGFPs expression vectors into *E.coli* and induced protein expression in the anaerobic chamber. The *E.coli* cells imaged in a standard green channel under confocal microscopy had easily detectable green fluorescence 20 h after protein expression induction (Figure 4A). Both miniGFPs were 2.3-fold brighter than phiLOV3 (Figure 4B) closely matching the relative brightness observed under normal oxygen concentration (Figure 1C). Thus, miniGFPs can be readily expressed and imaged under anaerobic conditions in *E. coli*.

**FIGURE 5:**
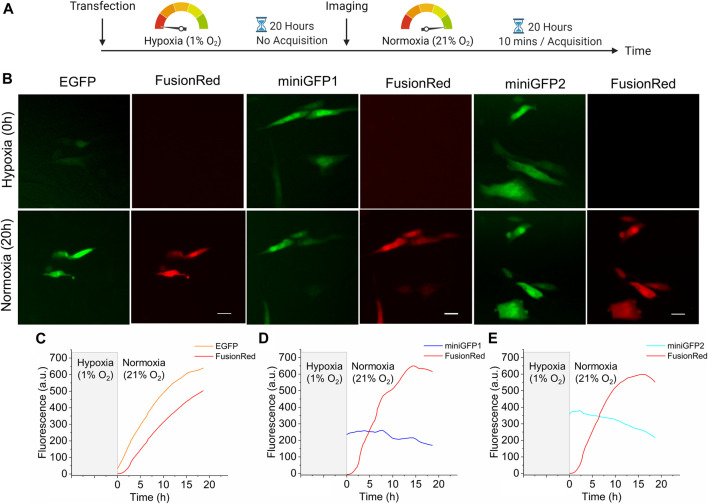
Characterization of miniGFPs under hypoxic conditions. **(A)** Experimental workflow of hypoxia experiments. **(B)** Fluorescence images of CHO cells expressing EGFP, miniGFP1 and miniGFP2 under hypoxia (upper row) and normoxia (lower row) conditions, respectively (for hypoxia images, cells after transfection were incubated under 1% oxygen, 10% carbon dioxide and 89% nitrogen condition for 20 h, for normoxia images, cells were incubated 20 h under 21% O2 atmosphere after hypoxia incubation). Scale bar, 20 µm. **(C–E)** The fluorescence changes of CHO cells co-expressing **(C)** EGFP, **(D)** miniGFP1, and **(E)** miniGFP2 with FusionRed under normoxia condition after 20 h of hypoxic incubation. (*n* = 10, 10, and 10 cells from 3, 2, 3 independent transfections for EGFP, miniGFP1, and miniGFP2, respectively).

**FIGURE 6:**
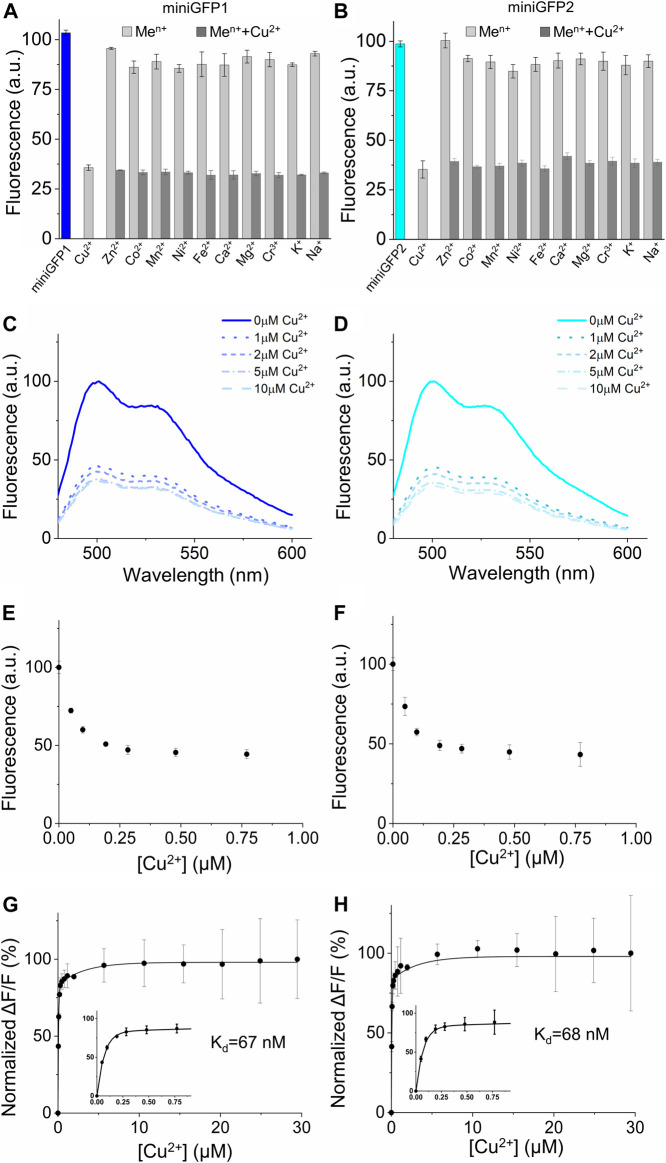
Metal sensitivity of miniGFPs in solution. **(A,B)** Selectivity and specificity of miniGFP1 and miniGFP2 to Cu^2+^ and other selected metal cations (*n* = 3 technical replicates each; column, mean, error bars, standard deviation). Data are normalized to the fluorescence intensity at ions-free conditions, pH 7.4. The measurements were performed at 100 μM for Cu^2+^, Zn^2+^, Co^2+^, Mn^2+^, Ni^2+^, Fe^2+^, at 800 μM for Ca^2+^, Mg^2+^, K^+^, Na^+^, and at 6 μM for Cr^3+^. miniGFP1 is shown as blue column and miniGFP2 is shown as cyan column, other metal cations are shown as light grey column. **(C,D)** Fluorescence spectra of miniGFP1 and miniGFP2 with increasing Cu^2+^ from 0 to 10 μM. Data are normalized to the fluorescence intensity at 500 nm in copper-free condition. **(E,F)** Titration of miniGFP1 and miniGFP2 shown as integrated fluorescence from plot **(C,D)** against Cu^2+^ concentration and normalized to initial fluorescence intensity. **(G,H)** The binding affinity curves of miniGFP1 and miniGFP2, the concentration range of the 0–0.75 μM Cu^2+^ are shown in the inset. Dots represent mean, error bars represent standard deviation for 3 technical replicates each.

Next, we evaluated the performance of miniGFPs under hypoxia conditions in CHO cells. To validate hypoxic conditions, miniGFPs were co-expressed with a bright red GFP-like FP, FusionRed (Shemiakina et al., 2012), which requires oxygen for the posttranslational cyclization to form a mature chromophore (Piatkevich et al., 2010). For reference, we also used the EGFP expression vector. Immediately after transfection, CHO cells were incubated under hypoxic conditions (1% O_2_, 10% CO_2_, 89% N_2_) for 20 h followed by live cell imaging under normoxia for 20 h (Figure 5A). Similar conditions were previously used for the evaluation of other oxygen-independent FPs without any observed negative affect on cell culture viability (Erapaneedi et al., 2016). After hypoxic incubation, the FusionRed fluorescence was not detectable while EGFP transfected cells exhibited green fluorescence only slightly above the background (Figure 5B). However, immediately after transferring cells into normoxic conditions, EGFP fluorescence started to increase reaching plateau in about 20 h (Figure 6C). The fluorescence of miniGFP1 and miniGFP2 were steady after recovering from hypoxia conditions and slightly decreased within 20 h period (Figures 6D,E). In all cases, FusionRed had about 2 h lag in fluorescence increase. Overall, these results demonstrated the applicability of miniGFPs for long-term live cell imaging under hypoxia conditions.

### Copper sensitivity of miniGFPs fluorescence

It was previously reported that fluorescence of some flavin-binding FPs can be quenched by transition metal ions, such as copper, zinc, and mercury with affinities around single micromolar values (Ravikumar et al., 2015a; Ravikumar et al., 2015b; Zou et al., 2020). In particular, the miniGFPs’ precursor iLOV demonstrated an inherent affinity to Cu(II) in solution with K_d_ of 4.72 μM (Ravikumar et al., 2015b). Therefore, we sought to determine the sensitivity of the miniGFPs fluorescence to various biologically relevant metal ions in solution using purified proteins. For the metal ion sensitivity assay, we chose multiple metals including alkali metals (potassium and sodium at 800 μM), alkaline Earth metals (magnesium and calcium at 800 μM), and transition metals (cobalt, zinc, iron, nickel, copper, manganese at 100 μM and chromium at 6 μM). By measuring fluorescence intensity before and after metal ion administration, we found that the most significant quenching of fluorescence was observed in the case of Cu^2+^ (by ∼65% for both miniGFPs; Figures 6A,B). In addition, Ni^2+^ ions quenched fluorescence of both miniGFPs by about 15%, while Co^2+^ ion decreased miniGFP1 fluorescence by 15%, which was slightly higher than that for miniGFP2 (Figures 6A,B). The rest of the tested ions showed only insignificant fluorescence response (∼10%) and did not interfere with copper sensitivity (Figures 6A,B).

To further characterized fluorescence quenching of miniGFPs, we performed titration in a wide range of Cu^2+^ concentrations from 0 to 30 μM. Administration of Cu^2+^ reduced fluorescence intensity however, the emission profiles were not altered (Figures 6C,D). The robust fluorescence response was observed at 50 nM of [Cu^2+^] and at [Cu^2+^] = 200 nM the fluorescence intensity was decreased by ∼50% reaching the maximum fluorescence quenching of 63% and 66% for miniGFP1 and miniGFP2 at 10 μM, respectively (Figures 6E–H). Thus, the estimated dynamic range of Cu^2+^ detection was from 10 nM to 10 μM. The normalized ∆F/∆F_max_ curves against copper concentration were fitted well to a single binding site model with K_d_ values of 67 nM and 68 nM for miniGFP1 and miniGFP2, respectively (Figures 6G,H).

Copper is a redox-active metal ion that can exist in two oxidation states in biological systems: Cu^+^ (reduced) and Cu^2+^ (oxidized) (Kim et al., 2008). Therefore, we also tested the sensitivity of miniGFPs to the reduced form of Cu, which is considered to be the predominant form in eukaryotic cells due to the reducing environment of cytoplasm (Blackburn et al., 2014). The fluorescence spectra were recorded against the increasing Cu^+^ concentration from 0 μM to 6.5 μM to quantify fluorescence intensity quenching (Supplementary Figure S5C). More than 10% of fluorescence quenching was detected at 0.9 µM of [Cu^+^] and at the highest tested [Cu^+^] the fluorescence of miniGFP1 and miniGFP2 dropped by 41% and 52%, respectively (Supplementary Figures S5A,B). Using a single binding site model, the estimated K_d_ of Cu^+^ for miniGFP1 and miniGFP2 were 2.2 and 2.5 μM, respectively (Supplementary Figures S5D,E). These results demonstrated that miniGFPs fluorescence was sensitive to both forms of Cu in solution with high selectivity. However, miniGFPs had almost two orders of magnitude higher affinity to Cu^2+^ than to Cu^+^ with a broader dynamic range, although the amplitudes of fluorescence quenching were comparable.

### Affinity of miniGFPs to flavins

**FIGURE 7:**
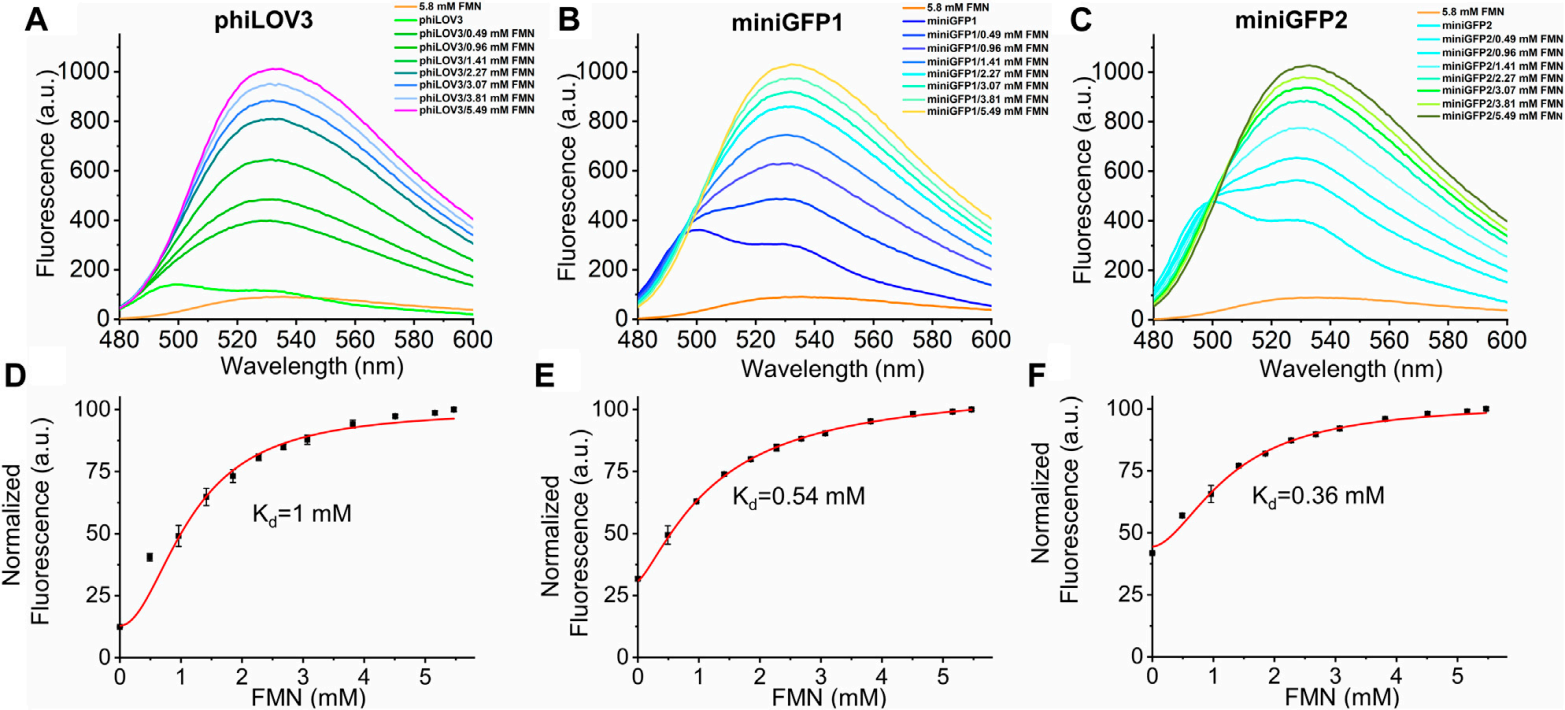
Spectral characterization of phiLOV3 and miniGFPs with an increasing concentration of exogenous FMN. **(A–C)** The fluorescence emission spectra of phiLOV3 and miniGFPs with addition of FMN at the concentration ranging from 0 to 5.49 mM. Compared to pure FMN (5.8 mM, orange line), the fluorescent spectra of phiLOV3 (green line) miniGFP1 (bule line) and miniGFP2 (cyan line) all changed after the introduction of FMN with the fluorescence increase. **(D–F)** The binding affinity curves of phiLOV3 and miniGFPs with FMN. The corresponding of estimated K_d_ values was calculated by the Hill 1 equation. Dots represent mean, error bars represent standard deviation for three technical replicates each.

Riboflavin and its derivatives, FMN and FAD, are the most common forms of flavins found in biological contexts (Giancaspero et al., 2015). It was reported that some flavin-binding proteins exhibited high affinity to multiple physiological flavins (Mathes et al., 2009; Anderson et al., 2020). We sought to explore affinity of *E. coli* expressed miniGFPs to flavins in solution. We first titrated different concentrations of FMN, FAD, and riboflavin to phiLOV3 and miniGFPs using aqueous solutions of the corresponding flavins as a blank control. Physiological flavins exhibit intrinsic fluorescence that can be used for their detection and analysis *in vitro* (Hühner et al., 2015; Galbán et al., 2016) therefore it was important to account for fluorescence of free flavins upon titration. Administration of FMN at a final concentration of 5.8 mM resulted in about 10-fold fluorescence intensity increase while titration of riboflavin and FAD resulted in quenching phiLOV3 and miniGFPs fluorescence (Suppementary Figure S6). We further characterized FMN binding by titrating aliquots of FMN into solutions containing phiLOV3 and miniGFPs and recording fluorescence emission. Binding of FMN altered spectral profiles by broadening the emission bands and shifting their peaks from ∼500 to ∼530 nm (Figures 7A–C). By fitting the fluorescence measurements to a binding model assuming noncooperative binding behavior we determined K_d_ of 0.54 and 0.36 mM for miniGFP1 and miniGFP2, respectively, while the estimated K_d_ for phiLOV3 was about 1 mM (Figures 7D–F). These results indicated that miniGFPs expressed in *E. coli* contained a significant fraction of the apoproteins that could bind exogenous FMN in solution although the formed adducts exhibited altered emission profiles. The miniGFP proteins exhibited a higher affinity to FMN than their precursor phiLOV3. From another hand, FAD and riboflavin did not show specific binding to miniGFPs or phiLOV3.

## Discussion

We reported the development and characterization of a pair of the enhanced flavin binding GFPs, called miniGFP1 and miniGFP2. To develop miniGFPs we employed directed molecular evolution, which is one of the most common approaches for FPs optimization (Subach et al., 2011). We chose to optimize brightness and photostability as the most important properties for live cell imaging. Moreover, flavin-binding FPs, in particular iLOV derivatives, are known to generate singlet oxygen species under blue light illumination, which can cause increased phototoxicity (Baier et al., 2006; Shu et al., 2011). Therefore, we suggested that photobleaching screening should also facilitate the selection of clones with lower phototoxicity since after illumination only live cells could further be cultured. Three rounds of directed evolution were sufficient to enhance the intracellular brightness in *E. coli* cells without significant alternation of spectral properties or stability of the template protein phiLOV3. Furthermore, as expected, the introduced mutations did not affect the ability of miniGFPs to fluorescence when expressed under anaerobic and hypoxic conditions. Moreover, when expressed in bacteria miniGFPs do not require co-expression of additional enzymes or administration of exogenous co-factors unlike other oxygen-independent FPs including bilirubin binding UnaG protein and biliverdin binding NIR FPs (Piatkevich et al., 2013; Chia et al., 2020), which simplifies miniGFPs applications. It is also important to note that miniGFPs’ performance was sufficient for long-term (∼20 h) live cell imaging accompanied by only minor photobleaching (20%–30%; Figure 5). Improved photostability and absence of dark recovery make miniGFPs particularly attractive for quantitative imaging in comparison with other bright flavin-binding FPs, such as CreiLOV and BR1, which can be considered as photoswitchable FPs. However, miniGFPs are still characterized by lower intracellular brightness and photostability than the most widely used GFP-like FP EGFP (Supplementary Figure S4) and therefore further optimization might be needed to widen their applicability.

It was previously reported that some flavin binding FPs exhibited the fluorescence sensitivity to Cu(II) in solution characterized by affinities in a single micromolar range (Ravikumar et al., 2015b; Zou et al., 2020). Interestingly, the estimated Cu(II) affinity for miniGFPs was almost two orders of magnitude higher than that reported for other flavin-binding FPs iLOV and CreiLOV (Ravikumar et al., 2015b; Zou et al., 2020). This could be due to the mutation in the putative metal binding motif (Supplementary Figure S2). As previously suggested, the Cu ion can be coordinated by Asn residues in the close vicinity of the FMN chromophore resulting in dynamic quenching of the fluorescence (Ravikumar et al., 2015b; Zou et al., 2020). As a possibility, the substitution of Asn by Tyr at position 15 in miniGFPs improved copper ion coordination, however, elucidation of the exact mechanism of Cu-sensitivity requires further functional and structural studies. The calculated K_d_’s for miniGFPs were comparable to one of the most sensitive Cu(II) biosensors engineered based on EGFP (16 nM for EGFP-based biosensor vs. 67–68 nM for miniGFPs) (Bálint et al., 2013), while the highest affinity to Cu(II) among all FPs was recently discovered in biliverdin binding protein miRFP670 reaching picomolar range (Zhao and Zastrow, 2022). From another hand, there was no literature data on flavin binding FP sensitivity to Cu(I), which represents more physiologically relevant form of copper in eukaryotic cells. Here, for the first time to our knowledge, we described sensitivity of flavin binding FPs to Cu(I) (Figure 6). However, it should be admitted that the estimated K_d_ for Cu(I) was not within the biologically relevant range of free Cu^+^ ions concentration. In general, despite significant progress in engineering fluorescence biosensors for Cu(I) and Cu(II) ions, none of the currently available biosensors were utilized for copper imaging in live cells under physiologically relevant conditions but rather under an artificially created environment with excessive concentration of copper salt in extracellular buffer complemented by pharmacological stimulation (Wegner et al., 2010; Castro et al., 2014; Zou et al., 2020). The difficulty of intracellular copper detection is most likely associated with an extremely low concentration of free ions (10^−17^−10^−21^ M) as majority of copper in cells is buffered by endogenous proteins and stored in a tightly bound, kinetically labile form (Wegner et al., 2011). Nevertheless, copper is the third most abundant heavy metal in humans, which is involved in a wide range of physiological processes primarily as a co-factor for metalloenzymes (National Research Council (US) Committee on Copper in Drinking Water, 2000; Tapiero et al., 2003). At the same time, the abnormal function of cellular copper homeostasis results in severe neurodegenerative diseases such as Alzheimer’s disease, etc. (Desai and Kaler, 2008). Therefore, optical biosensors for *in situ* copper imaging will be very important for further investigation of its biological function. However, currently available genetically encoded fluorescent probes with copper sensitivity would require additional optimization to meet the requirements. Intrinsic copper sensitivity of miniGFPs with nanomolar affinity makes them perspective templates for copper biosensor development. From another hand, adapting *in vitro* analysis of copper concentration is important for food safety, environmental pollution live monitoring, and diagnostics. The most popular assays for copper detection in soil and water samples are mainly based on spectrometry, such as UV-Vis spectrometry, atomic absorption spectrometry, and X-ray fluorescent spectrometer (Elkhatat et al., 2021). For example, to a typical Cu^2+^ level for surface water in China is ranging from 5 to 15 μM (Zhao et al., 2009). In this perspective, miniGFPs can find immediate application for fluorescence-based analysis of water pollution due to their nanomolar affinity and high specificity.

To further explore the utility of miniGFPs, we tested the ability of miniGFPs to detect flavin molecules including FMN, its precursor riboflavin, and another derivative, flavin adenine dinucleotide (FAD). For example, previously Anderson et al. reported the development of a fluorescence turn-on sensor for riboflavin based on the iLOV apoprotein (Anderson et al., 2020). In our hands, as observed by fluorescence spectra, miniGFPs purified from *E.coli* cells did not show any specific binding to riboflavin or FAD but administration of exogenous FMN significantly increased fluorescence. This might be due to a fraction of apoprotein, which was capable to bind FMN, however, we also observed changes in emission spectrum profile becoming more reminiscences of the pure FMN emission profile and might be a result of non-specific bind or protein aggregation. Elucidation of the exact mechanism of FMN binding will require further studies and goes outside of the scope of the current studies. As detection of FMN and other flavin derivatives in biological fluids is used for diagnostics of some diseases, this feature of miniGFPs might create a basis for express methods of FMN analysis in clinic.

## Conclusion

We developed a pair of small GFPs, named miniGFPs, and performed their systematic characterization *in vitro* and in different mammalian cell cultures in comparison with other bright GFPs. The miniGFP proteins are characterized by improved photophysical properties compared to other bright flavin-binding GFPs that allowed their utilization for long-term live cell culture imaging under normoxia and hypoxia. *In vitro* sensitivity of miniGFPs to copper ions and exogenous FMN makes them suitable starting templates for biosensor development, however, the molecular mechanism of copper and FMN sensitivity requires further studies.

## Experimental procedures

### Molecular cloning and directed evolution

The UnaG and FusionRed genes were synthesized *de novo* by GenScript, based on the sequences reported in the original publications (Shemiakina et al., 2012; Kumagai et al., 2013). The phiLOV3 and EGFP genes were PCR amplified from pphiLOV3-N1 (Addgene plasmid #178973) and pEGFP-N1 (Clontech) plasmids, respectively. Synthetic DNA oligonucleotides used for cloning were purchased from Healthy Creatures (Hangzhou, China). PrimeStar Max master mix (Clontech) was used for high-fidelity PCR amplifications. Restriction endonucleases were purchased from (New England Biolabs, United States) and used according to the manufacturer’s protocols. Small-scale isolation of plasmids was done with QIAGEN plasmid mini kit by QIAcube connect (QIAGEN, German). Sequencing was performed by the Sanger method (Healthy Creatures Hangzhou, China). Molecular cloning was performed using NovoRec plus one step Cloning kit (Novoprotein, China). Error-prone PCR of the phiLOV3 genes was performed using Mutazyme II DNA polymerase (Agilent, United States) under high mutation rate conditions (9–16 mutations per kilobase pair) and subcloned into the pBAD-HisD vector. The generated libraries were electroporated in TOP10 cells (Tsingke, China) and expressed in LB media supplemented with ampicillin (Amp^+^: 100 mg/μl) and 0.02% L-arabinose (Ara) for 24 h at 37°C. For quality control, after electroporation an aliquote (1 µl) of each library was plated on LB/agar plates to estimate the library size and confirm ligation efficiency and mutation rate by sequencing 20 randomly picked colonies. All generated libraries contained 1–10 × 10^6^ independent clones with three to four nucleotide mutations per gene. Bacterial cultures expressing the libraries were illuminated with 450 nm using a custom assembled LED array (Lot#997-LXK0-PR04-0008, Mouser Electronics) for 5 min and sorted using FACS (BD FACS Melody, United States). The collected cells were plated on LB/agar plates supplemented with Amp^+^ and Ara and incubated overnight. The colonies were imaged before and after 450 nm illumination with LED in a green channel: (Ex: 460–495 nm; Em: 510–550 nm) using an SZX16 fluorescence stereomicroscope (Olympus, Japan) equipped with Spectra III Light Engine (LumenCor, United States) and cooling color digital camera (BGIMAGING, China). The selected colonies were cultured in LB medium containing Amp^+^ and Ara in 24-well deep well plates, treated with B-PER (Thermo Fisher, United States) and analyzed using a fluorescent plate reader (Varioskan LUX, Thermo Fisher Scientific, United States). For expression in mammalian cell lines, the target plasmids were cloned in two steps. First, EBFP2 in pAAV-CAG-EBFP2-P2A-GFP (Addgene plasmids #184938) was swapped with the gene of parental protein-phiLOV3, and genes of miniGFP1, miniGFP2, EGFP, and UnaG using *BamH*I and *Age*I sites. Then, GFP was swapped with FusionRed using *Spe*I and *EcoR*I sites.

### Protein purification and *in vitro* characterization

Proteins were expressed in the TOP10 strain (Biomed, China) using pBAD-HisD vector and purified as previously described (Babakhanova et al., 2022). The collected protein solutions were dialyzed overnight against PBS and stored at 4°C for more than 6 months without noticeable degradation or spectral properties changes. The protein concentration was determined by the BCA protein quantification kit (Yeasen, China). The absorption spectra were measured by UV-Vis-NIR UV3600Plus Spectrophotometer (Shimadzu, Japan) and the fluorescence spectra were measured by using FS5 Spectrofluorometer (Edinburgh Instruments, United Kingdom). The lifetime and quantum yield were measured using an FLS1000 spectrometer equipped with the integrating sphere accessory (Edinburgh Instruments, United Kingdom) according to the manufacture protocol. The extinction coefficient was measured by the TCA denaturation protocol previously described (Chapman and Reid, 1999). For pH titration, the purified proteins were diluted in pH buffers (Hydron, United States) and fluorescence was recorded using Varioskan LUX Plate reader (ThermoFisher, United States). Thermostability was measured using qPCR machine (CFX connect, BioRAD, United States) by heating protein solutions at 0.2°C per minute till 90°C.

### Protein characterization under anaerobic conditions

1NEB Stable *E.coli* (NEB, United States) transformed with pBAD-HisD vectors containing phiLOV3, miniGFP1, and miniGFP2 were cultured in low salt LB medium anaerobically at 37°C for 20 h with the induction of 0.2% Ara. The glass slides containing the bacteria were made in the anaerobic chamber and sealed with nail polish. Images were captured with an upright confocal microscope at 488 nm with a 500–600 nm emission range (×60 objective, Olympus FV3000). Intensities of the green fluorescence were quantified using the ImageJ software.

### Protein characterization in mammalian cells

All procedures involving mice were conducted following the United States National Institutes of Health Guide for the Care and Use of Laboratory Animals and approved by the Westlake University Committee on Animal Care. MEF, HEK293, CHO, and HeLa (ATCC, United States) cell lines were cultured in DMEM (Servicebio, China) with 10% fetal bovine serum (FBS, Yeasen, China), supplemented with penicillin and streptomycin at 37°C and 5% CO_2_. Hieff Trans™ Liposomal Transfection Reagent kit (Yeasen, China) or Lipo3000 (Invitrogen) was used for transient transfection. For hypoxia conditions, CHO cells were cultured in the hypoxystation H35 (Don Whitley Scientific, United Kingdom) under an atmosphere of 1% O_2_, 10% CO_2_, and 89% N_2_ at 37°C. The C57BL/6J strain (supplied by the animal facility of Westlake University) was used for primary neuronal culture preparation regardless of sex. Neuronal culture preparation and transfection were carried out as previously described (Piatkevich et al., 2018; Papadaki et al., 2022). Intracellular brightness and photostability measurements were carried out under Nikon Ti2-E widefield microscope equipped with Spectra III Light Engine (LumenCore), the ORCA-Flash 4.0 V3 sCMOS camera (Hamamatsu), and 20X/0.75 objective lens controlled by NIS Elements software using GFP (E×475/28 nm, Em 535/46 nm) and RFP (Ex 555/28 nm, Em 594/40 nm) channels. For assessment of CreiLOV and BR1 brightness and photostability, prior to imaging the cells were kept in darkness for several minutes, and the first image was acquired in green channel with a short exposure (100 ms) to minimize photobleaching.

### Metal cations and flavins binding measurements

For metal cations selectivity experiments, fluorescent proteins were diluted to 30 μM with PBS and stock solutions of salts were added at final concentrations of 6, 100, or 800 μM. The stock solutions for ion titration were prepared by dissolving the salts in pure water as follows: 800 mM for CuSO_4_, ZnCl_2_, CoCl_2_·6H_2_O, MnCl_2_, NiCl_2_·6H_2_O, Fe(NH_4_)_2_SO_4_, and 100 mM for CaCl_2_, MgSO_4_, Cr(NO_3_)_3_·9H_2_O, KCl, NaCl. The binding curves were fitted in OriginPro software with Eq. 1 assuming a single metal-binding site in the protein based on previously reported results for other Cu-sensitive flavin-binding FPs (Ravikumar et al., 2015b), where [Cu] is the total copper concentration in the respective buffer and [P] is the concentration of miniGFPs. As a measure of the affinity for Cu binding, the Kd is concluded from the fitting curve when ΔF/ΔF_max_ reached 50% as follows (Eli and Chakrabartty, 2006; Ravikumar et al., 2015b; Zou et al., 2020):

$$\frac{\Delta F}{\Delta F_{\max}}=\frac{K_{d}+\left[P\right]+\left[Cu\right]-\sqrt{{\left(K_{d}+\left[P\right]+\left[Cu\right]\right)}^{2}-4\left[P\right]\left[Cu\right]}}{2\left[P\right]}$$ (1)

For copper(Ⅰ) titration, tetrakis (acetonitrile) copper(Ⅰ) hexafluorophosphate was used to prepare a metal stock of 100 μM in acetonitrile. Each 30 μM fluorescent protein was titrated with final working concertation of copper(Ⅰ) ranging from 0 to 6.5 μM. The binding curves with the Hill1 equation were fitted in OriginPro software. The flavins titration, riboflavin, FMN, and FAD (Sigma Aldrich, United States) were diluted in PBS at 25 μM, 25 mM and 25 mM, respectively, for the stock solutions (pH 7.4). The riboflavin stock solution was supplemented with 0.1 mM sodium hydroxide to improve solubility. For metal ions and flavins titration, fluorescence spectra were recorded by exciting at 450 nm, the emission spectra were scanned from 480 to 600 nm with the step of 1 nm using a fluorescence plate reader. The data of three flavins in PBS at working concentration were recorded for background correction.

### Image and data analysis

The data were processed by Nikon AR/BR Elements, Microsoft Excel, OriginPro 2019, PyMOL, and ImageJ. ANOVA-Fisher test was done by OriginPro 2019.

## Data Availability

The sequences for miniGFP1 and miniGFP2 are deposited in the GenBank databases (accession numbers OK323369 and OK323370, respectively). The plasmids used in this study are available from Addgene. All the experimental data are available upon request from the corresponding author.
