# Supplementary material for: Enhanced small green fluorescent proteins as a multisensing platform for biosensor development
Source: Front Bioeng Biotechnol. 2022 Oct 17;10:1039317. doi: 10.3389/fbioe.2022.1039317 (PMC9618808; doi:10.3389/fbioe.2022.1039317)
Supplement: Supplementary file 1 [file DataSheet1.docx]

**SUPPORTING INFORMATION**

**Enhanced small green fluorescent proteins as a multisensing platform for biosensor development**

Guo-Teng Liang^1,2,3,4^, Cuixin Lai^1,2,3^, Zejun Yue^1,2,3,5^, Hanbin Zhang^1,2,3^, Danyang Li^1,2,3^, Zhong Chen^6^, Xingyu Lu^6^, Liang Tao^1,2,3^, Fedor V. Subach^7^, Kiryl D. Piatkevich^1,2,3*^

^1^School of Life Sciences, Westlake University, Hangzhou, Zhejiang, China

^2^Westlake Laboratory of Life Sciences and Biomedicine, Hangzhou, Zhejiang, China

^3^Institute of Basic Medical Sciences, Westlake Institute for Advanced Study, Hangzhou, Zhejiang, China

^4^Department of Applied Biology and Chemical Technology, The Hong Kong Polytechnic University, Kowloon, Hong Kong, China

^5^School of Basic Medical Sciences, Xi'an Jiao Tong University, Xi’an, Shaanxi, China

^6^Key Laboratory of Precise Synthesis of Functional Molecules of Zhejiang Province, School of Science, Instrumentation and Service Center for Molecular Sciences, Westlake University, Hangzhou, Zhejiang, China

^7^Complex of NBICS Technologies, National Research Center “Kurchatov Institute”, Moscow, Russia

Correspondence to: [kiryl.piatkevich@westlake.edu.cn](mailto:kiryl.piatkevich@westlake.edu.cn)

**Table of contents**

| Item | Content | Page |
| --- | --- | --- |
| Figure S1 | Photostability selection of phiLOV3 mutants in bacterial libraries | S-3 |
| Figure S2 | The miniGFPs predicated structures and amino acid alignment with their precursors | S-4 |
| Figure S3 | Expression of miniGFP1 and phiLOV3 in cultured neuron | S-6 |
| Figure S4 | Characterization of miniGFPs in comparison to phiLOV3, EGFP, and UnaG in cultured mammalian cells | S-7 |
| Figure S5 | The sensitivity of miniGFPs to copper(I) | S-9 |
| Figure S6 | Evaluation of flavin-binding abilities of miniGFPs and phiLOV3 | S-10 |

**Figure S1.** Photostability selection of the phiLOV3 mutants in bacterial libraries.


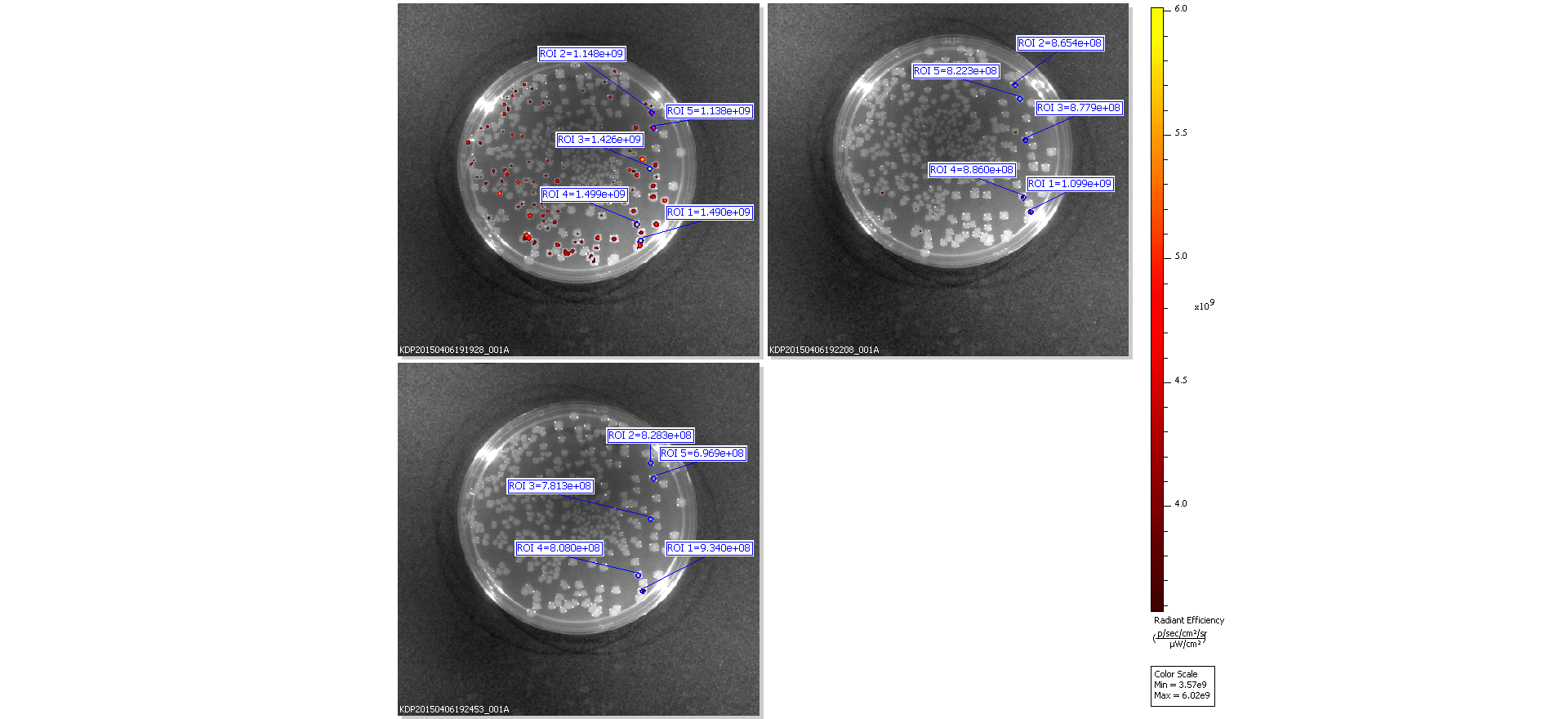

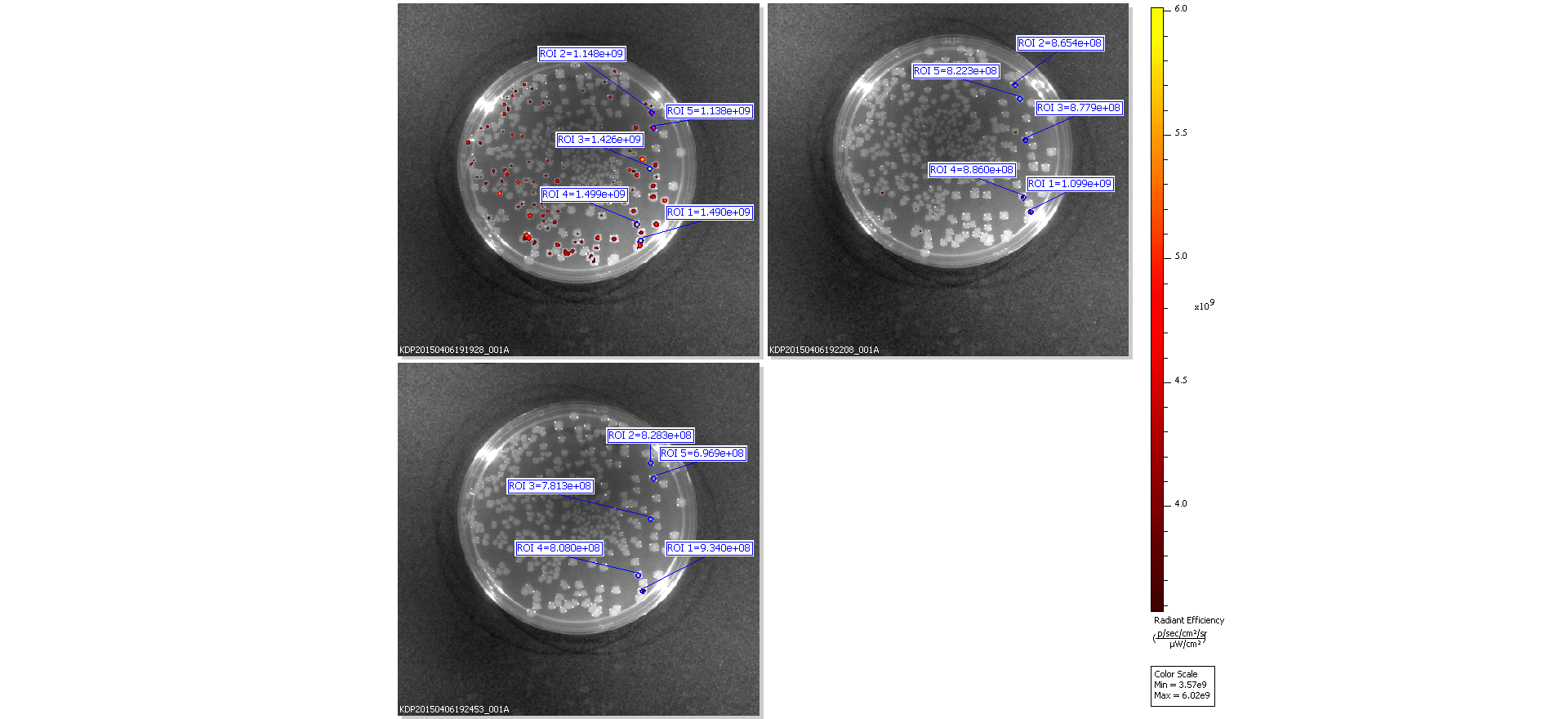

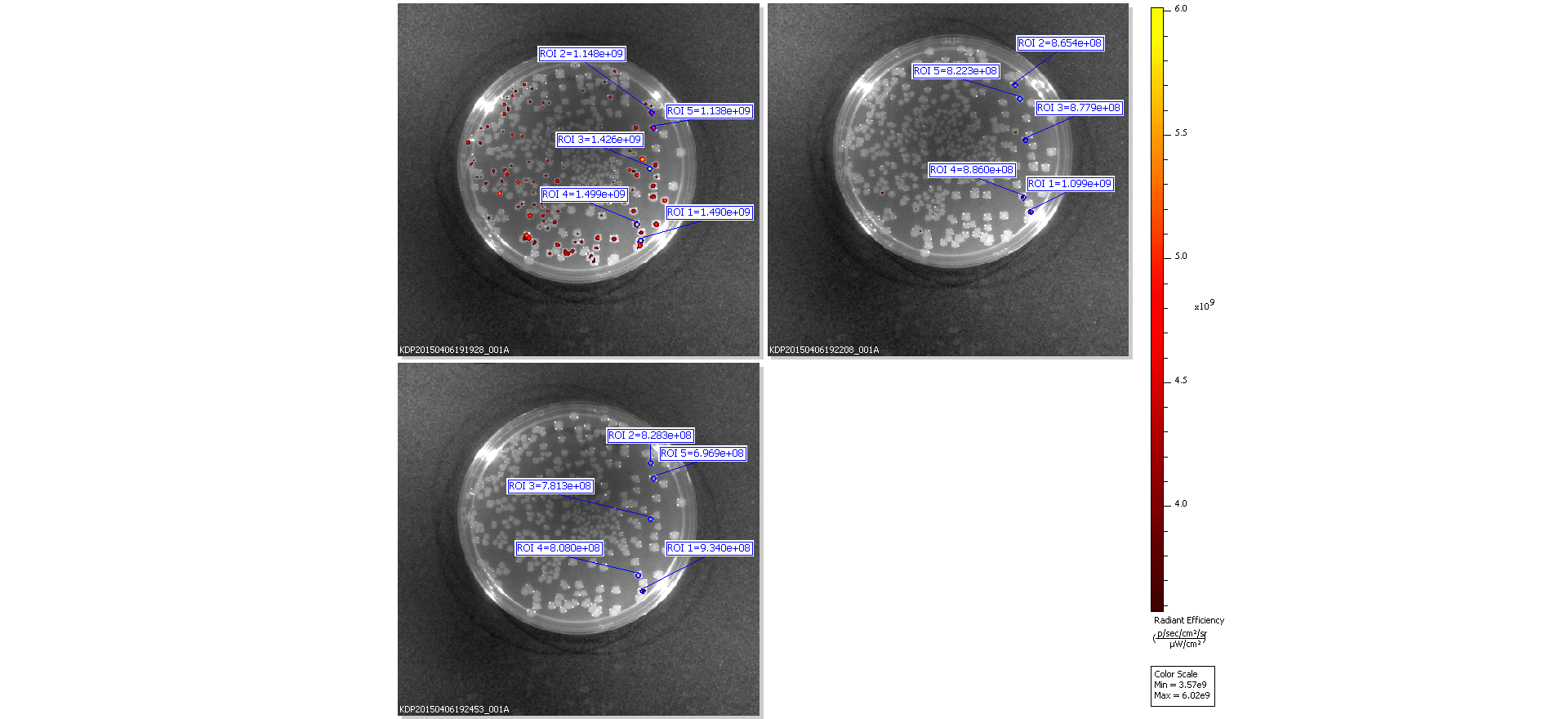

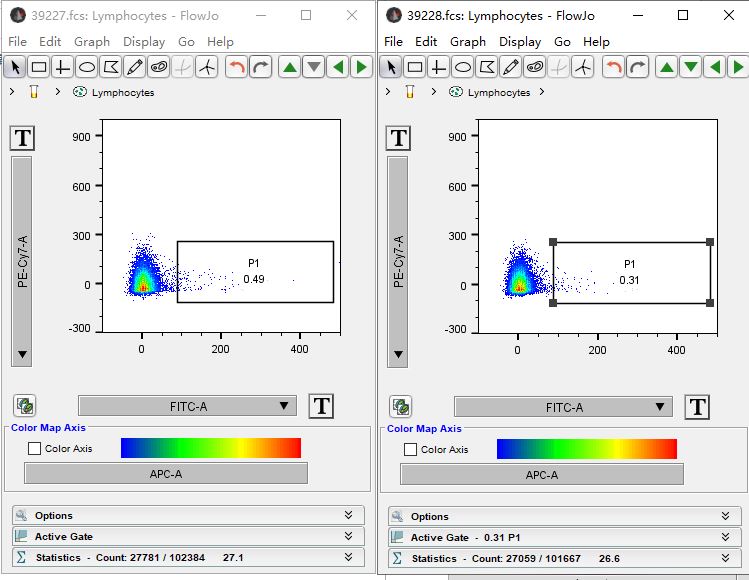

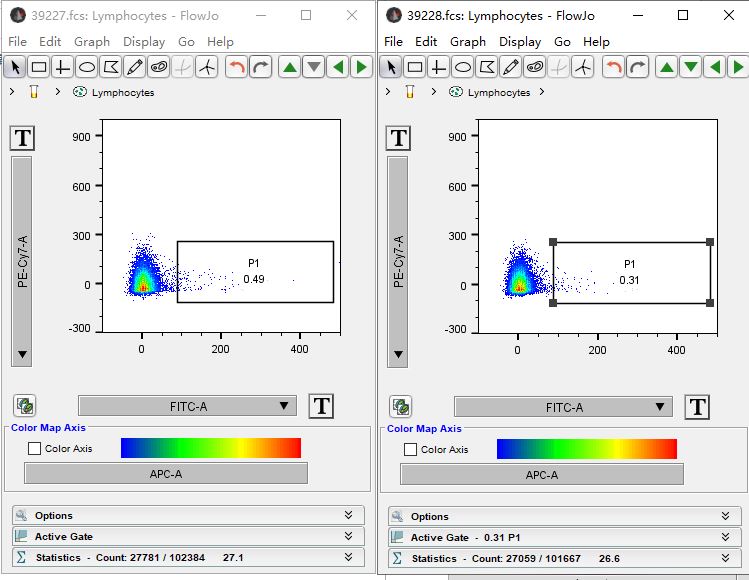


**A**

**B**

Ex: 488 nm; Em: 515/20 nm

Ex: 488 nm; Em: 515/20 nm

Non-illuminated

Illuminated

Before illumination

After illumination

Radiant Efficiency

4.0

4.5

5.5

5.0

6.0

x10^9^

(**A**) FACS dot-plots for non-illuminated (*left*) and illuminated (*right*) bacterial library expressing phiLOV3 mutants. The bacterial library was illuminated by 450 nm light from LED in PBS suspension for 5 min. (**B**) Bacterial colonies expressing phiLOV3 mutants selected with FACS before illumination (*left*) and after illumination (*right*) with 450 nm light. The labeled colonies were selected for spectroscopic measurements in solution using a fluorescence microplate.

**Figure S2.** The miniGFPs predicated structures and amino acid alignment with their precursors.


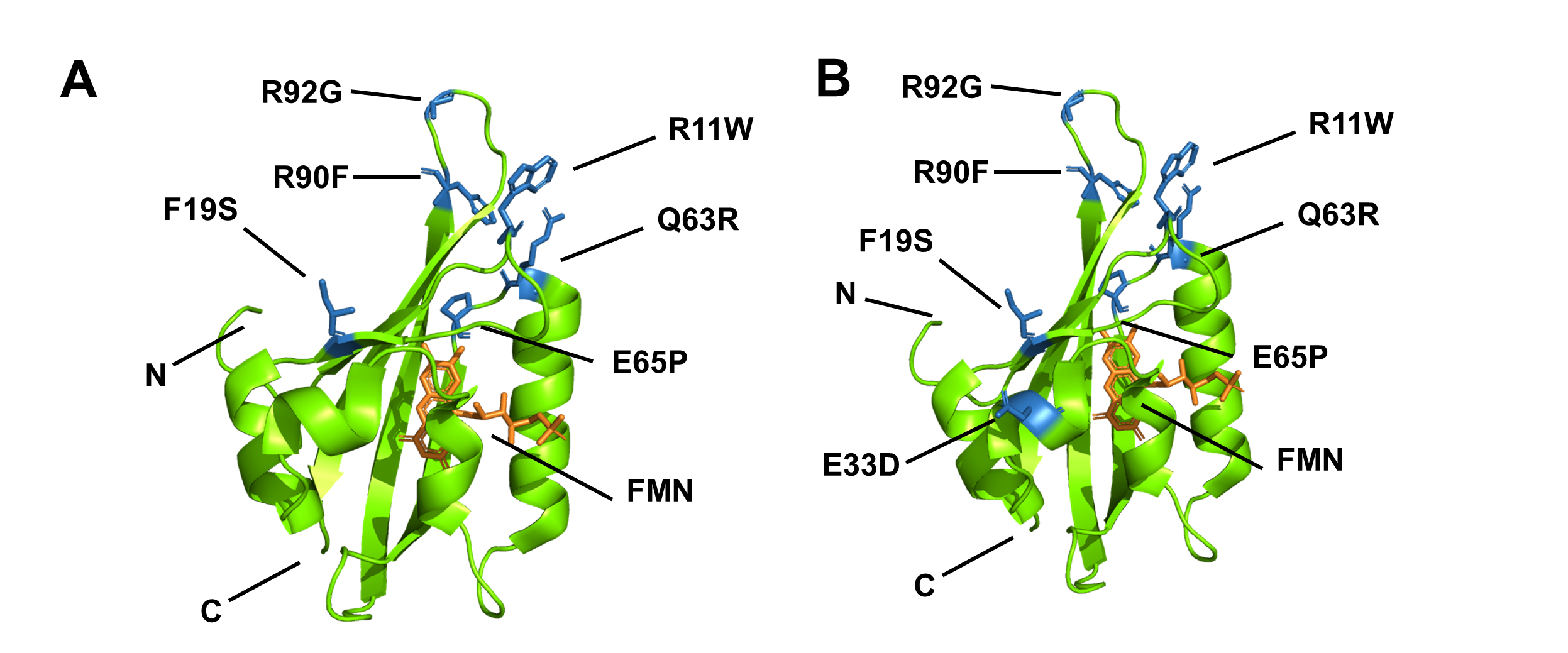


**C**

**10 20 30 40 50 60**

**| * | | *| | |**

**CreiL MAGLRHTFVVADATLPDCPLVYASEGFYAMTGYGPDEVLGHNARFLQGEGTDPKEVQKIRDAI**

**iLOV MIEKNFVITDPRLPDNPIIFASDGFLELTEYSREEILGRNARFLQGPETDQATVQKIRDAI**

**BR1 MIEKNYVITDPRLPDNPIIFASDGFLELTGYSREEILGRNARFLQGPETDQATVQKIRDAI**

**phiLOV2.1 MEKSFVITDPRLPDYPIIFASDGFLELTEYSREEIMGRNARFLQGPETDQATVQKIRDAI**

**phiLOV3 MEKSFVITDPRLPDYPIIFASDGFLELTEYSREEIMGRNARFLQGPETDQATVQKIRDAI**

**phiLOV3.1 MEKSFVITDPRLPDYPIISASDGFLELTEYSREEIMGRNARFLQGPETDQATVQKIRDAI**

**miniGFP1 MEKSFVITDPWLPDYPIISASDGFLELTEYSREEIMGRNARFLQGPETDQATVQKIRDAI**

**miniGFP2 MEKSFVITDPWLPDYPIISASDGFLELTEYSRDEIMGRNARFLQGPETDQATVQKIRDAI**

**
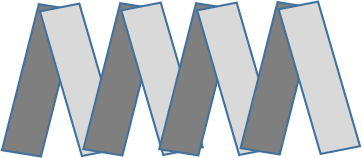

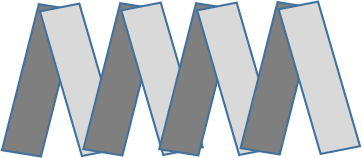

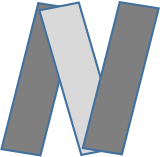

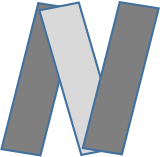
**

**70 80 90 100 110**

**| | | | |**

**CreiL KKGEACSVRLLNYRKDGTPFWNLLTVTPIKTPDGRVSKFVGVQVDVTSKTEGKALA**

**iLOV RDQRETTVQLINYTKSGKKFWNLLHLQPVRDQKGELQYFIGVQLDGSDHV**

**BR1 RDQRETTVQLINYTKSGRKFWNLLHLQPVRDQKGELQYFIGVQLDGSDRV**

**phiLOV2.1 RDQRETTVQLINYTKSGKKFWNLLHLQPVRDRKGGLQYFIGVQLVGSDHV**

**phiLOV3 RDRRETTVQLINYTKSGKKFWNLLHLQPVRDGKGGLQYFIGVQLVGSDHV**

**phiLOV3.1 RDRRPTTVQLINYTKSGKKFWNLLHLQPVFDGKGGLQYFIGVQLVGSDHV**

**miniGFP1 RDRRPTTVQLINYTKSGKKFWNLLHLQPVFDGKGGLQYFIGVQLVGSDHV**

**miniGFP2 RDRRPTTVQLINYTKSGKKFWNLLHLQPVFDGKGGLQYFIGVQLVGSDHV**

**
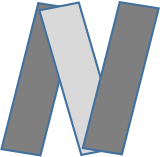
**

(**A,B**) The 3D protein structures of (**A**) miniGFP1 and (**B**) miniGFP2 predicted based on the phiLOV2.1 crystal structure (PDB:4EEU). The FMN chromophore is shown in orange and the mutated amino acids are shown in blue. (**C**) Amino acid sequence alignment of CreiLOV (CreiL), iLOV, BR1, phiLOV2.1, phiLOV3, phiLOV3.1, miniGFP1, and miniGFP2. Mutations resulting in the conversion of phiLOV2.1 into evolved downstream proteins are highlighted in cyan. Amino acids highlighted in green are the residues surrounding the chromophore within 3.5 Å. The β-sheet-forming regions and α-helixes are shaded and illustrated with arrows and ribbons, respectively. The amino acids marked with asterisk are suggested to coordinate copper ions. The sequences for miniGFP1 and miniGFP2 are deposited in the GenBank databases (accession numbers OK323369 and OK323370, respectively). The plasmids used in this study are available from Addgene.

**Figure S3.** Expression of miniGFP1 and phiLOV3 in cultured hippocampal mouse neurons.

**
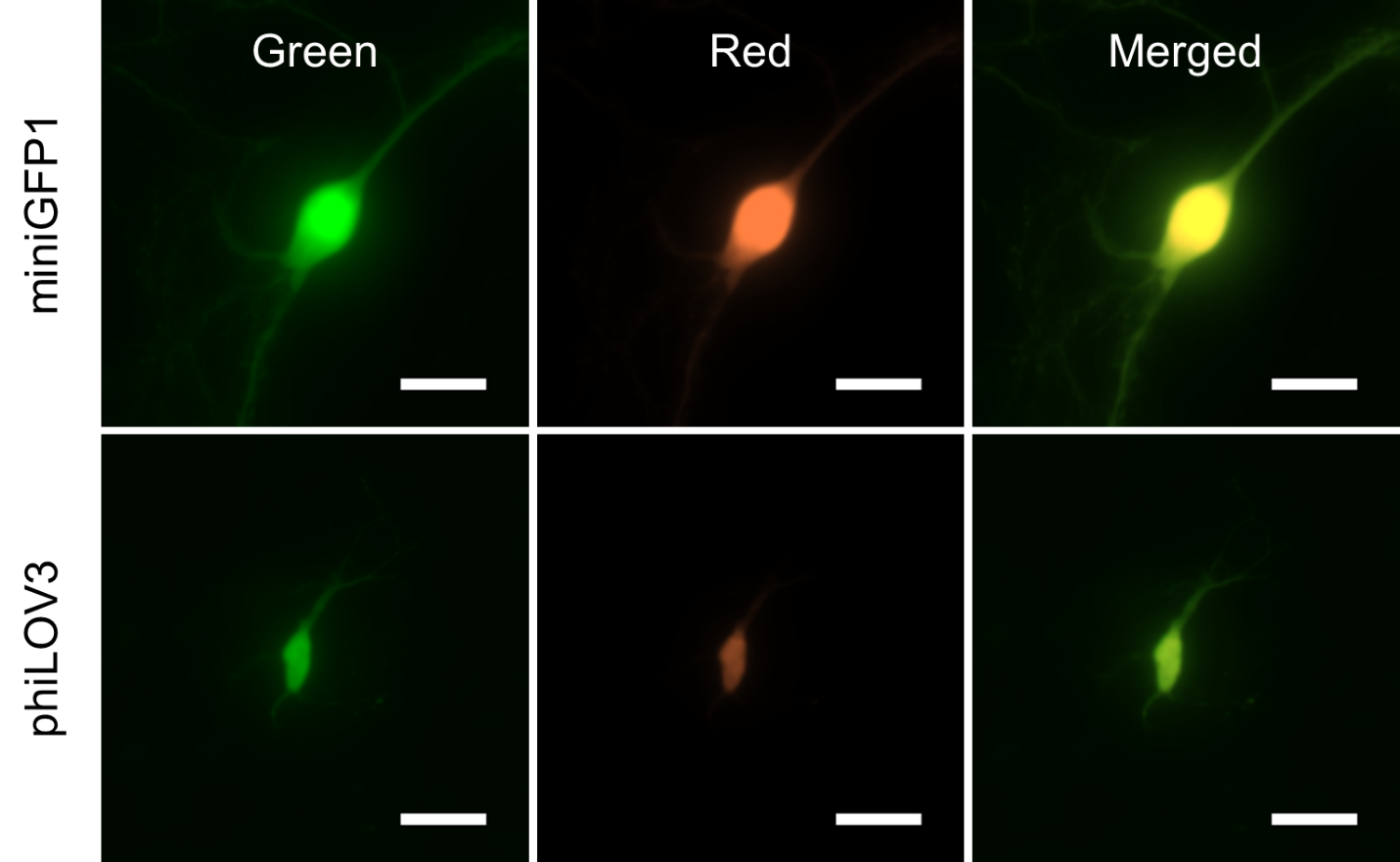
**

Representative fluorescence images obtained from cultured neurons transfected with pAAV-CAG plasmid encoding green FP (miniGFP1or phiLOV3) and red reference FP FusionRed. The images were adjusted to the same LUT for visualization. Imaging conditions: 475/28 nm excitation, 535/46 nm emission for miniGFPs and phiLOV3; 555/28 nm excitation, 594/40 nm emission for FusionRed. Scale bar, 20 µm.

**Figure S4.** Characterization of miniGFPs in comparison to phiLOV3, EGFP, and UnaG in cultured mammalian cells.


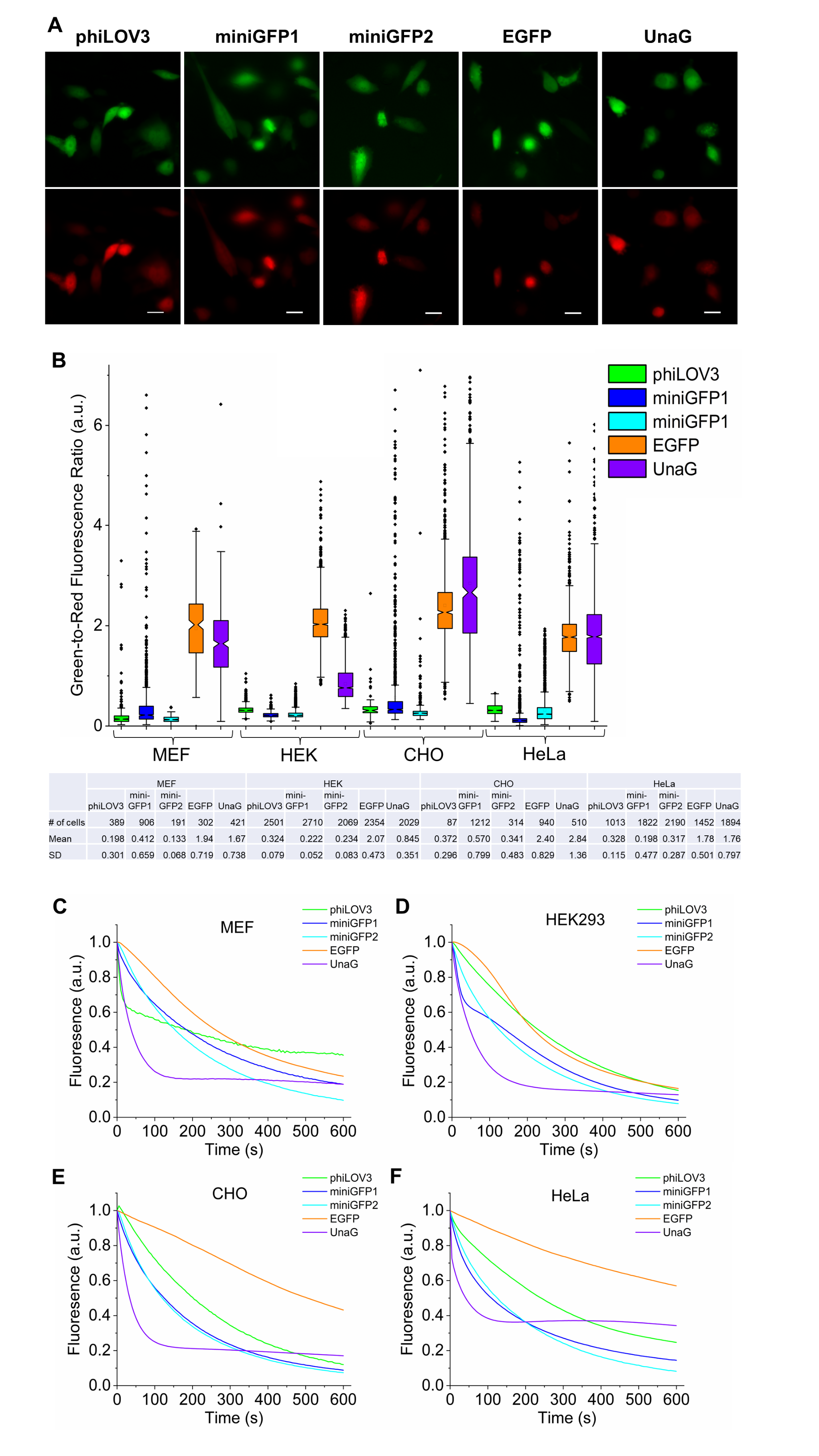


The phiLOV3, miniGFP1, miniGFP2, EGFP, and UnaG protein were transiently co-expressed with FusionRed via the P2A peptide using pAAV-CAG vector in cultured mammalian cells for about 36 h and imaged under wide-field epifluorescence microscope. (**A**) Representative images of CHO cells expressing the selected proteins in green and red channels. Imaging conditions: 475/28 nm excitation, 535/46 nm emission for green FPs; 555/28 nm excitation, 594/40 nm emission for FusionRed. Scale bar, 20 µm. (**B**) Green-to-red fluorescence ratios for the selected FPs expressed in MEF, HEK, CHO, and HeLa cells (statistics shown in the table below; # of cells, number of cells from 2 independent transfections each; mean, mean value of fluorescence ratio; SD, standard deviation; box plots with notches are used, narrow part of notch, median; top and bottom of the notch, 95% confidence interval for the median; top and bottom horizontal lines, 25% and 75% percentiles for the data; whiskers extend 1.5× the interquartile range from the 25th and 75th percentiles; horizontal line, mean; dots, outliers). Imaging conditions are the same as in a. (**C,D,E,F**) Photobleaching curves for the selected proteins recorded in MEF, HEK, CHO, and HeLa cells under continuous wide-field illumination (475/28 nm).

**Figure S5.** The sensitivity of miniGFPs to copper(I).

**
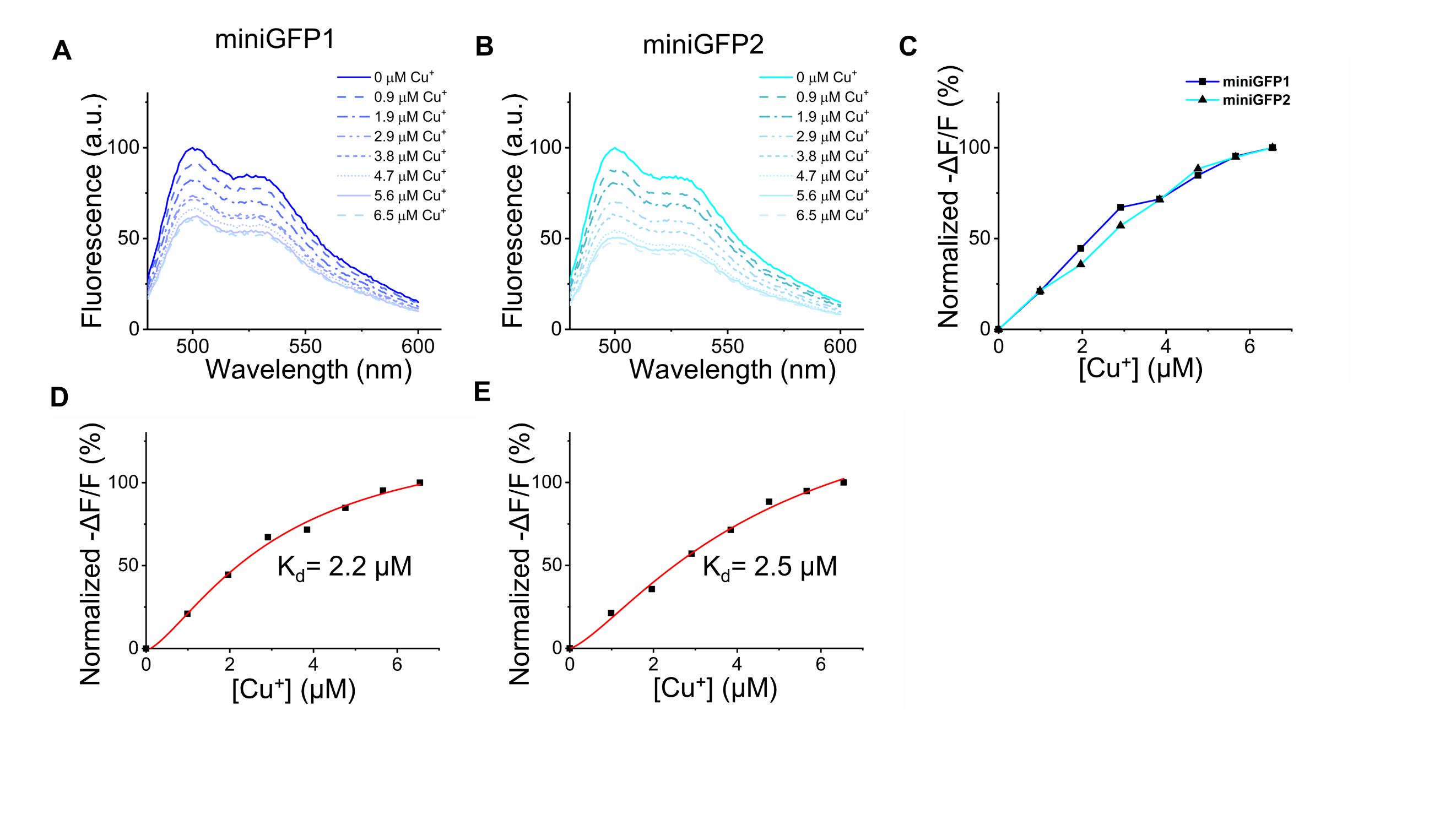
**

(**A,B**) The fluorescence emission spectra of miniGFP1 and miniGFP2 in the presence Cu^+^ in the range of concentration from 0 μM to 6 μM. (**C**) The normalized fluorescence changes of miniGFP1 and miniGFP2 at the increasing [Cu^+^] at final concentration of 6 μM. Data are normalized to final fluorescence intensity, the data points of miniGFP1 and miniGFP2 represent in square and up triangle, respectively (**D,E**) .The fitting curves (red line) of miniGFP1 and miniGFP2 binding affinity, the corresponding K_d_ values were calculated by Hill and Hill1 equations, respectively.

**Figure S6.** Evaluation of flavin-binding abilities of phiLOV3 and miniGFPs in solution.


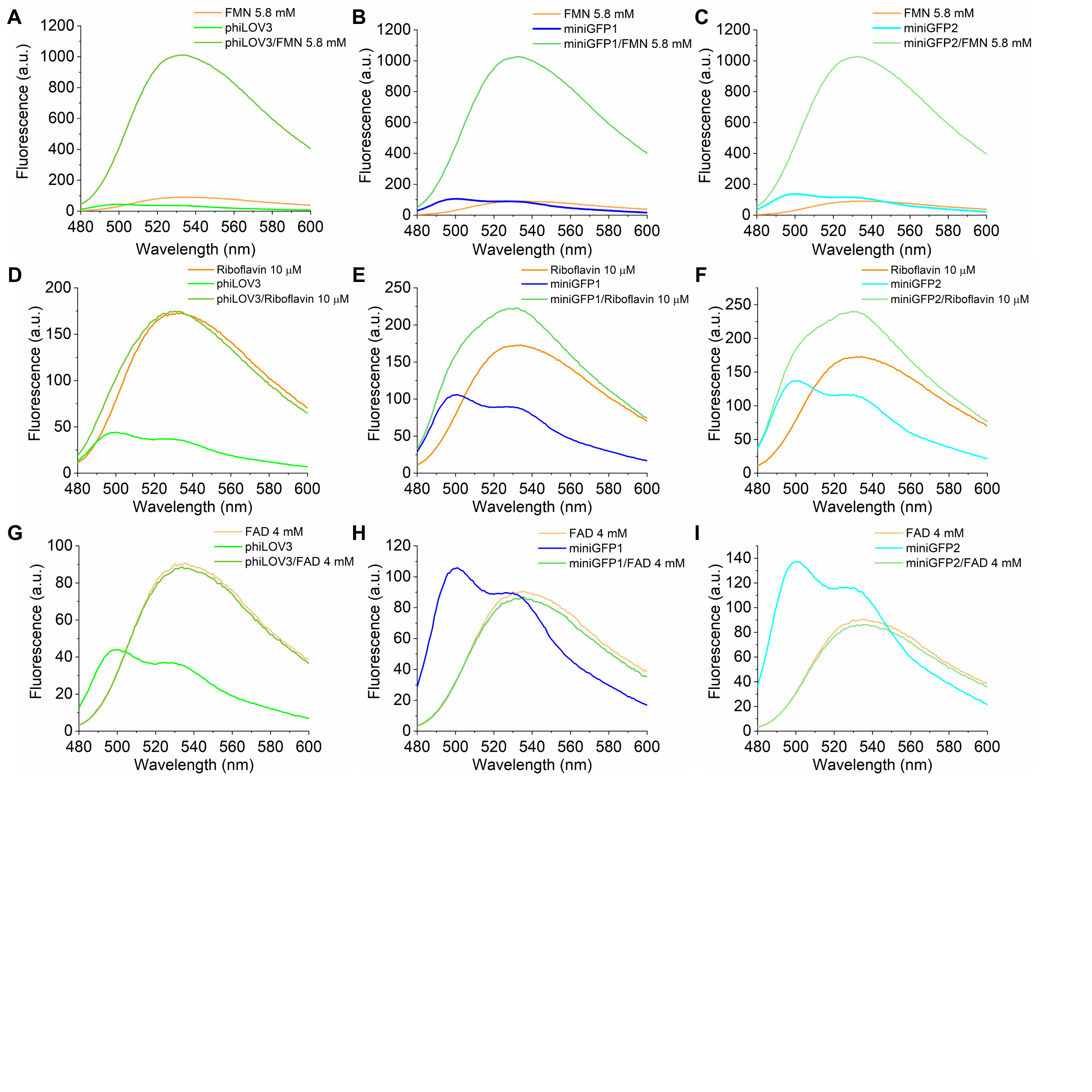


(**A**,**B**,**C**) Fluorescence spectra of free 5.8 mM FMN in PBS, *E. coli* expressed miniGFPs and phiLOV3, and FPs in 5.8 mM FMN. (**D**,**E**,**F**) Fluorescence spectra of free 10 μM riboflavin in PBS, *E. coli* expressed miniGFPs and phiLOV3, and FPs in 10 μM riboflavin. (**G**,**H**,**I**) Fluorescence spectra of free 4 mM FAD in PBS, *E. coli* expressed miniGFPs and phiLOV3, and FPs in 4 mM FAD.
